# Immobilized Black Radish Peroxidase on ZnO‐Alginate Beads: A Multifunctional Biocatalyst for Sustainable Food Packaging

**DOI:** 10.1002/fsn3.70694

**Published:** 2025-08-20

**Authors:** Songul Bayrak

**Affiliations:** ^1^ Faculty of Science, Department of Chemistry Ataturk University Erzurum Turkiye

**Keywords:** antimicrobial, antioxidant, black radish peroxidase, calcium alginate, food packaging, immobilization, zinc oxide

## Abstract

This study presents a novel bioactive food packaging material developed by immobilizing black radish peroxidase (Br‐POD) calcium alginate beads loaded with zinc oxide nanoparticles (ZnO/CA). The immobilization process achieved an efficiency of 90.6% and increased enzyme‐specific activity by 36%. The ZnO/CA‐Br‐POD composite exhibited enhanced thermal stability (optimal temperature shift from 40°C to 60°C) and pH tolerance (from pH 6 to 5), along with strong antimicrobial activity against *Staphylococcus aureus* and *Escherichia coli* . Antioxidant performance, evaluated via DPPH and ABTS radical scavenging assays, showed up to 93.4% activity. Application on Agaricus bisporus mushrooms reduced weight loss by 49.5% during storage, demonstrating significant preservation capacity. These findings underscore the potential of ZnO/CA‐Br‐POD composites as multifunctional, eco‐friendly materials for active food packaging applications.

## Graphical abstract

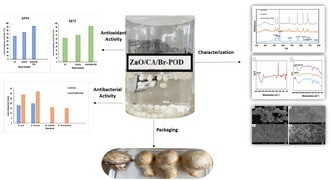

## 1 Introduction

In recent years, the food industry has shown increasing interest in active packaging systems that extend shelf life and prevent microbial spoilage. Edible films and coatings have emerged as promising strategies for preserving food quality and reducing postharvest losses (Lahlali et al. 2020; Soliva‐Fortuny and Martín‐Belloso 2003). The incorporation of bioactive agents, such as natural antioxidants and antimicrobials, into these materials has garnered considerable attention due to their potential to enhance food safety and quality (Deshmukh and Gaikwad 2024).

Antioxidants are defined as molecules that inhibit the oxidation of other molecules. From a food perspective, antioxidant compounds are known to significantly delay or completely halt the oxidation of an oxidizable substrate, even at low concentrations (Gulcin and Alwasel 2022). Antioxidants help reduce cellular damage and maintain overall health by preventing oxidative damage in foods and living organisms. In addition to inhibiting lipid peroxidation, antioxidants can slow the progression of many chronic diseases (Yazıcıoğlu et al. 2025; Gulcin 2025). In basal metabolism, the primary endogenous elements of these defense mechanisms include enzymes, such as superoxide dismutase (SOD), peroxidase (POD), and glutathione reductase (GR), as well as antioxidant molecules, such as glutathione, α‐tocopherol, and ascorbic acid (Bayrak et al. 2024; Gulcin 2020).

In addition, synthetic antioxidants (e.g., BHA, BHT, TBHQ) are widely used in food systems to prevent the oxidative degradation of food products. However, concerns regarding their potential toxic and carcinogenic effects have prompted regulatory restrictions and fueled the search for safer, natural alternatives (Erdoğan et al. 2021; Polat Kose and Gulcin 2021). In this context, regulatory bodies have imposed restrictions on synthetic antioxidants, prompting a search for safer, natural alternatives (Bingol et al. 2021). Enzymes are increasingly being considered as a promising solution in this regard (Alotaibi et al. 2025).

Enzymes are essential catalysts that accelerate chemical reactions in biological systems. Compared with many traditional methods in food and pharmaceutical applications, enzymes offer advantages such as low toxicity, biodegradability, milder reaction conditions, low energy costs, and protection (Öztürk et al. 2022; Weng et al. 2023).

Peroxidases (EC 1.11.1.7) are among the most widely used enzymes in environmental applications. They constitute a vital class of antioxidant enzymes found ubiquitously in nature (Oztekin et al. 2019). Their primary functions include catalyzing the reactions between compounds that can donate hydrogen atoms and hydrogen peroxide (H_2_O_2_), which acts as an acceptor for these molecules. The rapid removal of H_2_O_2_, an oxidizing agent produced during the metabolic processes of living organisms, is crucial for maintaining cellular integrity and preventing oxidative damage (Zuleyha Almaz et al. 2021; Gerni and Özdemir 2024). Antioxidant enzymes such as peroxidase efficiently manage this detoxification process within cells. These enzymes effectively break down H_2_O_2_ and convert it into harmless byproducts via various phenolic and nonphenolic substrates (Züleyha Almaz 2023).

Owing to their versatility in catalyzing redox reactions on various substrates, peroxidases have broad applications in multiple fields, including medicine, biochemistry, immunology, biotechnology, food shelf‐life extension, and industry.

However, the practical use of soluble enzymes poses challenges, including low stability, high cost, lack of reusability, and difficulty in recovering them from reaction mixtures. Immobilization techniques have emerged as effective strategies to overcome these limitations. Immobilization helps retain enzyme activity over repeated use, enhances thermal and operational stability, and allows easier separation and reusability of enzymes. Enzyme immobilization can be achieved through various physical or chemical techniques, including adsorption, covalent bonding, entrapment, and encapsulation.

Immobilization preserves enzymatic activity during reuse, reduces production costs, and enhances catalytic stability and tolerance to adverse conditions (Deshmukh and Gaikwad 2024). Organic and inorganic supports, which do not dissolve in the reaction medium, can be used for enzyme immobilization (Matto and Husain 2009).

In particular, entrapment of enzymes within calcium alginate matrices is an economical, non‐toxic, and efficient immobilization approach. Alginate offers high biocompatibility and gel‐forming capacity in the presence of divalent cations such as Ca^2+^. Moreover, combining alginate with inorganic nanoparticles such as zinc oxide (ZnO) results in hybrid nanocomposites with enhanced mechanical strength and functional performance. ZnO nanoparticles possess unique optical, chemical, and antimicrobial properties, making them valuable components in bioactive packaging systems. Although ZnO is an inorganic material, it is considered generally recognized as safe (GRAS) by the FDA in small concentrations for certain food applications. (Zyoud et al. 2023).

Combining calcium alginate with ZnO nanoparticles (ZnO/CA) creates an effective hybrid support. Alginate is cost‐effective, nontoxic, biodegradable, and compatible with enzymes, while ZnO NPs enhance mechanical resistance and reduce enzymatic leakage, a common issue in peroxidase immobilization.

Various enzyme immobilization methods have been widely used to improve the operational stability and reusability of enzymes in practical applications (Alatawi et al. 2020; Mohamed et al. 2004).

In this study, black radish ( *Raphanus sativus* ) peroxidase (Br‐POD) was immobilized into ZnO‐incorporated calcium alginate (ZnO/CA) beads to form a stable biocatalytic system with antioxidant and antimicrobial properties. These ZnO/CA‐Br‐POD beads were applied to Agaricus bisporus mushrooms to investigate their effects on extending shelf life and preventing weight loss. Mushrooms are highly perishable due to their high water content and metabolic activity. The use of ZnO/CA‐Br‐POD beads aims to create a protective microenvironment through the slow release of bioactive agents, thereby delaying spoilage and preserving the product's quality. This application not only serves as a model for evaluating the potential of immobilized enzyme systems in active packaging but also contributes to the development of sustainable postharvest preservation technologies.

## 2 Materials and Methods

### 2.1 Materials

Black radish (*Raphanus sativus L*.) and mushrooms (*Agaricus bisporus*) were obtained from a local market in Erzurum, Turkey. They were subsequently stored at −80°C in a deep freezer until required for the study. The chemicals employed in this research, including the sulfonamide, zinc nitrate hexahydrate [Zn (NO_3_)_2_·6H_2_O], sodium hydroxide (NaOH), guaiacol, hydrogen peroxide (H_2_O_2_), calcium chloride (CaCI_2_), sodium alginate, α‐tocopherol, BHA, BHT, Trolox, DPPH•, ABTS, and DMPD, were procured from Sigma Aldrich Company.

### 2.2 Crude Extract and Purification of Br‐POD Enzyme

Approximately 20 g of black radish was ground using liquid nitrogen and homogenized in 0.3 M phosphate buffer (pH 7.0) using ULTRA‐TURRAX (IKA, Staufen, Germany). The homogenate was centrifuged at 20,000 ***g*** for 30 min at 4°C. The supernatant was collected and stored at −80°C. For purification, affinity chromatography was performed using a 10 mL CNBr‐activated Sepharose 4 B‐sulfanilamide affinity gel column equilibrated with 20 mM phosphate buffer (pH 7.0). After the sample application, unbound proteins were removed, and the enzyme was eluted using acetate buffers (pH 5.5) containing 0.5–1 M NaCl. Active fractions were dialyzed and stored at −80°C (Gerni and Özdemir 2024).

### 2.3 Protein Determination for Free and Immobilized Peroxidase

Protein concentration was measured using the Bradford (1976), employing bovine serum albumin (BSA) as the standard, as outlined in the studies by Bayrak et al. (2020). Absorbance was recorded at 595 nm using a UV–Vis spectrophotometer (Shimadzu UV‐1800, Kyoto, Japan). The binding of protein to the immobilized substrate was evaluated by examining the filtrate and the wash solutions (Bayrak et al. 2020; Bradford 1976).

### 2.4 Free and Immobilized Peroxidase Activity Assay

The enzymatic activity of free and immobilized Br‐POD was determined spectrophotometrically by monitoring the oxidation of guaiacol to tetraguaiacol at 470 nm. The reaction mixture consisted of 8 mM guaiacol, 4 mM H_2_O_2_, 0.2 M phosphate buffer (pH 7.0), and 10 μL of enzyme solution. One unit of activity was defined as the amount of Br‐POD required to catalyze the oxidation of 1 μmol of guaiacol per minute (Lavery et al. 2010).

The EU is calculated via the formula below.

$$\frac{\mathrm{EU}}{\mathrm{mL}}=\frac{∆\mathrm{OD}}{\epsilon .t}.\frac{\mathrm{VT}}{\mathrm{VE}}.\mathrm{SF}$$

EU: Enzyme unit, ΔOD: Absorbance change (in 3 min), ε: extinction coefficient, SF: dilution factor (for diluted samples), VT: total cuvette volume, VE: volume of enzyme present in the cuvette, t: time.

### 2.5 Sodium Dodecyl Sulfate Polyacrylamide Gel Electrophoresis (SDS‐PAGE)

After the chromatographic procedure, SDS‐PAGE was used to control the purity of the BR‐POD enzyme, following previous procedures (Köksal et al. 2019). A single protein band was observed at a molecular weight of 44 kDa.

### 2.6 Synthesis and Characterization of Materials

#### 2.6.1 Synthesis of Zinc Oxides (ZnO)

ZnO nanoparticles were synthesized using a modified co‐precipitation method. A solution of 1 M zinc nitrate was stirred, and 2 M NaOH was added dropwise to maintain a pH of 10–11, resulting in a color change to pale yellow. The mixture was stirred at 45°C for 4 h, and the precipitates were precipitated, washed, dried at 110°C for 5 h, and calcined at 500°C for 2 h to obtain ZnO NPs (Abo‐Dief et al. 2022).

#### 2.6.2 Preparation of Calcium Alginate and ZnO/CA Beads

Blank alginate beads were produced by adding a 2 mL solution of 3% low‐viscosity sodium alginate in deionized water to a 25 mL beaker containing a stirred 100 rpm solution of 2.5% calcium chloride. The alginate solution was administered dropwise from a 5 mL syringe (0.8 × 38 mm) at a height of 3 cm.

To fabricate empty magnetic alginate beads, a total of 10 mg of ZnO was added to 2 mL of low‐viscosity sodium alginate solution prepared in distilled water (3%), followed by 5 min of ultrasonication. The resulting mixture was then gently dispensed into 20 mL of CaCl_2_ solution (2.5%) in a 25 mL beaker, which was stirred at 100 rpm and immersed in water. This was achieved using a syringe (5 mL; 0.8 × 38 mm). The production process was carried out by dropping the mixture from a height of 3 cm (Batool et al. 2024). The alginate solution had a pH of approximately 7.0 and was maintained at room temperature (22°C–25°C) throughout the bead formation process.

#### 2.6.3 Immobilization of Br‐POD on the ZnO/CA Nanocomposite

For BR‐POD immobilization, 1 mL of 0.4 mg/mL BR‐POD solution was mixed with 2 mL of sodium alginate and stirred at 100 rpm for 5 min. The immobilization was conducted at 25°C ± 1°C, and the pH of the alginate solution was adjusted to 7.0 to ensure enzyme integrity. For immobilization with ZnO, 5 mg of ZnO was ultrasonicated (Hielscher Ultrasonics GmbH, Teltow, Germany) in 2 mL of sodium alginate for 5 min, followed by the addition of BR‐POD solution and stirring for an additional 5 min. The resulting solutions were added to 20 mL of 2.5% CaCl_2_ solution with a syringe (5 mL, 32 mm) from a height of 3 cm (100 rpm), and immobilization was carried out by trapping the enzymes (Yildiz Dalginli et al. 2024). The immobilized beads were stored in 0.2 M phosphate buffer (pH 7.0) at 4°C for subsequent analysis.

The effectiveness of immobilization was calculated via the following formula:

- Specific activity (EU/mg).
- Specific activity of free enzyme (SAFE).
- Specific activity of the immobilized enzyme (SAIE).
- Immobilization yield (IY)
$$\%\mathrm{IY}=\frac{\left(\text{SAIE}\right)}{\left(\text{SAFE}\right)}\times 100$$

### 2.7 Optimization of the Immobilization Conditions

Peroxidase activities were examined at different concentrations of ZnO, sodium alginate, and calcium chloride to optimize the immobilization conditions according to a standard protocol. The immobilization procedure with ZnO was repeated with 3, 6, and 10 mg concentrations to optimize the ZnO concentration. Other variables were held constant, and various concentrations of calcium alginate (1.5%, 2.0%, 2.5%, and 3.0%) and CaCl_2_ (1.5%, 2.0%, 2.5%, and 3.0%) were used to optimize the enzyme concentration by adding Br‐POD solutions (0.4 mg/mL). Thus, three different injectors were used to determine the optimum enzyme concentration (0.60 × 32 mm, 0.70 × 32 mm, and 0.80 × 38 mm). The diameter of the beads was measured with a caliper. An average of 10 beads was used to determine the optimum bead size (Oleyaei et al. 2016).

### 2.8 Biochemical Characterization of Free and Immobilized Br‐POD

#### 2.8.1 Structural Characterization

FTIR spectra were recorded using a PerkinElmer Spectrum Two FT‐IR spectrometer (PerkinElmer Inc., Waltham, MA, USA) in the 400–4000 cm^−1^ range. Surface morphology was analyzed by field emission scanning electron microscopy (FESEM) using a ZEISS GeminiSEM 500 (Carl Zeiss AG, Oberkochen, Germany), and elemental composition was determined by energy‐dispersive X‐ray spectroscopy (EDS) with an Oxford Instruments X‐Max detector. X‐ray diffraction (XRD) patterns were recorded using a Bruker D8 Advance X‐ray diffractometer (Bruker Corp., Billerica, MA, USA) between 10° and 80° (2θ) (Shariat et al. 2018).

#### 2.8.2 Biochemical Characterization

##### 2.8.2.1 Effects of pH and Temperature on the Activity and Stability of Free and Immobilized Peroxidase

The activity of free and immobilized Br‐POD was determined over a pH range of 4.0 to 10.0. Reactions were carried out at 25°C for 30 min using the following buffer systems:

- 50 mM acetate buffer for pH 4.0, 5.0, and 6.0.
- 200 mM phosphate buffer for pH 6.0, 6.5, 7.0, and 7.5.
- 50 mM Tris–HCl buffer for pH 8.0 and 9.0.
- 50 mM glycine‐NaOH buffer for pH 10.0.

Enzyme activity was measured spectrophotometrically at 470 nm by monitoring the oxidation of guaiacol. The relative activity at each pH value was calculated by setting the highest activity as 100% (Abd Rahim et al. 2013; Gulmez et al. 2018).

For pH stability, both free and immobilized enzymes were pre‐incubated in the respective buffer systems across the same pH range (4.0–10.0) at their optimal temperature (40°C for free, 60°C for immobilized Br‐POD) for 60 min. After incubation, residual enzyme activity was measured under standard assay conditions.

For temperature activity profiling, enzyme solutions were incubated at various temperatures: 30°C, 40°C, 50°C, 60°C, 70°C, and 80°C, each at their optimal pH for 30 min. The relative activity was expressed as a percentage of the maximum observed activity.

To assess thermal stability, both enzyme forms were incubated at each of the above temperatures for 1 h, and the residual activity was again measured.

##### 2.8.2.2 Reusability of the Immobilized Peroxidase

The reusability of the immobilized enzyme was evaluated by repeatedly using the same alginate beads in enzymatic reactions according to the standard activity protocol until the enzymatic activity decreased to a negligible level. After each reaction cycle, the remaining activity of the immobilized enzyme was calculated, and the initial activity (100%) was used as a control.

##### 2.8.2.3 Storage Stability of Enzymes

The storage stability of free and immobilized enzymes was determined by measuring their activities after 1, 7, and 14 days under three different storage conditions: 4°C and 25°C. The enzymatic activity measured at the beginning of the storage period was taken as 100%.

### 2.9 Antioxidant Activity of Prepared Films

#### 2.9.1 CA and ZnO/CA/Br‐POD Beads DPPH Radical Scavenging Assay

The ability of CA and ZnO/CA/Br‐POD beads to scavenge free radicals was evaluated using the stable DPPH radical. For this, 25 mg of the beads were immersed in 4 mL of a 0.1 mM DPPH solution prepared in methanol and incubated at room temperature in the dark for 30 min. Incubation was carried out at room temperature (22 C–25 C), and the initial pH of the reaction medium was adjusted to approximately 7.0 for maximum peroxidase activity (Ton‐That et al. 2025). The absorbance of the resulting supernatant was recorded at 517 nm using UV–Visible spectroscopy, and the DPPH radical scavenging activity was determined:

$$\text{DPPH radical scavenging activity}\left(\%\right)=\frac{\text{Acontrol}-\text{Asample}}{\text{Acontrol}}\times 100$$

Acontro, Absorbance value of DPPH/methanol solution; Asample, Absorbance value of the bead mixture.

#### 2.9.2 CA and ZnO/CA/Br‐POD Beads ABTS Radical Scavenging Assay

For the ABTS assay (radical cation decolorization assay), a solution was prepared by combining 7 mM ABTS with 2.45 mM potassium persulfate in a 1:1 ratio, followed by incubation in the dark for 16 h. The beads were then immersed in 20 mL of this solution and allowed to react in the dark at room temperature for 30 min. Incubation was carried out at room temperature (22°C–25°C), and the initial pH of the reaction medium was adjusted to approximately 7.0 for maximum peroxidase activity (He et al. 2025). The absorbance of the supernatant was measured at 734 nm using UV–Visible spectroscopy, and the ABTS radical scavenging activity was subsequently determined:

$$\text{ABTS radical scavenging activity}=\frac{\text{Acontrol}-\text{Asample}}{\text{Acontrol}}\times 100$$

Acontrol, absorbance value of the ABTS solution, Asample; absorbance value of the bead mixture.

### 2.10 Agar Well Diffusion Assays of Prepared Films

The antimicrobial activity of CA, ZnO, and ZnO/CA/Br‐POD beads was evaluated using the method by Almaz (2023). Two gram‐positive bacteria (*Streptococcus pyogenes ATCC 19615 and Staphylococcus aureus ATCC 25923*) and two gram‐negative bacteria (*Escherichia coli O157 and Bacillus cereus ATCC 14579*) were inoculated onto Mueller‐Hinton Agar (MHA) medium at 37°C. After spreading the culture on agar plates, 10‐mm wells were created, and a total of 25 mg of beads was placed into the wells. Inhibition zone diameters were measured, and the results were compared with control CAs.

### 2.11 Evaluation of Films as Packaging Materials for Mushroom (*Agaricus Bisporus*) Preservation

The shelf life of mushrooms covered with CA, ZnO/CA, and ZnO/CA/Br‐POD, respectively, was studied following minor modifications to the procedure by Kumar et al. (2018). Mushrooms weighing 6–8 g, with no visible defects, were used (Kumar et al. 2018).

The samples were covered in four different conditions, such as (1) open air (control), (2) CA beads, (3) ZnO/CA beads, and (4) ZnO/CA/Br‐POD beads.

The mushrooms were stored in sterile containers, and sensory evaluation was done daily over 9 days by visually inspecting their external appearance. Weight loss was measured with a digital balance (±0.001 g), and daily weight loss was calculated using a specific formula.

$$\text{Weight lost}\;\left(\%\right)\frac{\mathrm{mo}-m}{\mathrm{mo}}\times 100$$

where mo represents the initial mass (g), and where m corresponds to the mass at 8 days (g).

### 2.12 Statistical Analysis

All data were analyzed using two‐way Analysis of Variance (ANOVA), and differences were considered statistically significant at *p* < 0.05.

## 3 Results and Discussion

### 3.1 Purification Steps of Br‐POD

**FIGURE 1:**
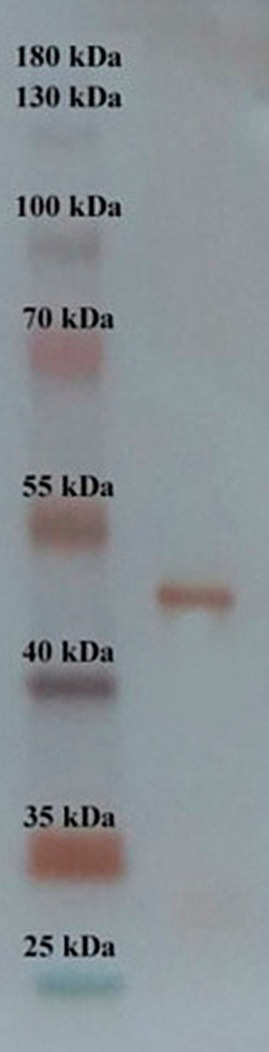
SDS‐PAGE image showing a single band at ~44 kDa, indicating the purity of the Br‐POD enzyme isolated from black radish via affinity chromatography.

The peroxidase enzyme was obtained from black radish with 35.97% yield and 66263.15 U/mg specific activity (601.13‐fold) via a rapid and efficient one‐step affinity technique using CNBr‐activated Sepharose‐4B‐L‐tyrosine‐sulphanilamide. The purification results are shown in Table 1. SDS‐PAGE, a primary method for accurately purifying the Br‐POD enzyme, was performed under denaturing conditions. The molecular weight of the Br‐POD enzyme obtained from the affinity gel was calculated to be approximately 44 kDa. The SDS‐PAGE image is shown in Figure 1.

**TABLE 1:** Purification results of Br‐POD enzyme using CNBr‐activated Sepharose‐4B‐L‐tyrosine‐sulphanilamide affinity chromatography, including total protein, activity, specific activity, yield, and purification fold at each step.

Purification steps	Total volume (mL)	Activity (EU/mL)	Protein (mg/mL)	Total activity (EU)	Total protein (mg)	Specific activity (EU/mg)	Yield %	Purification fold
Homogenate	5	120	1.10	600	5.5	110.23	100	1
Sepharose‐4 B‐L‐Tyrosine‐sulphanilamide affinity chromatography	2	110,9	0.0015	220.4	0.003	73933.33	36.73	670.71

### 3.2 Synthesis of the Zinc Oxides (ZnO)

**FIGURE 2:**
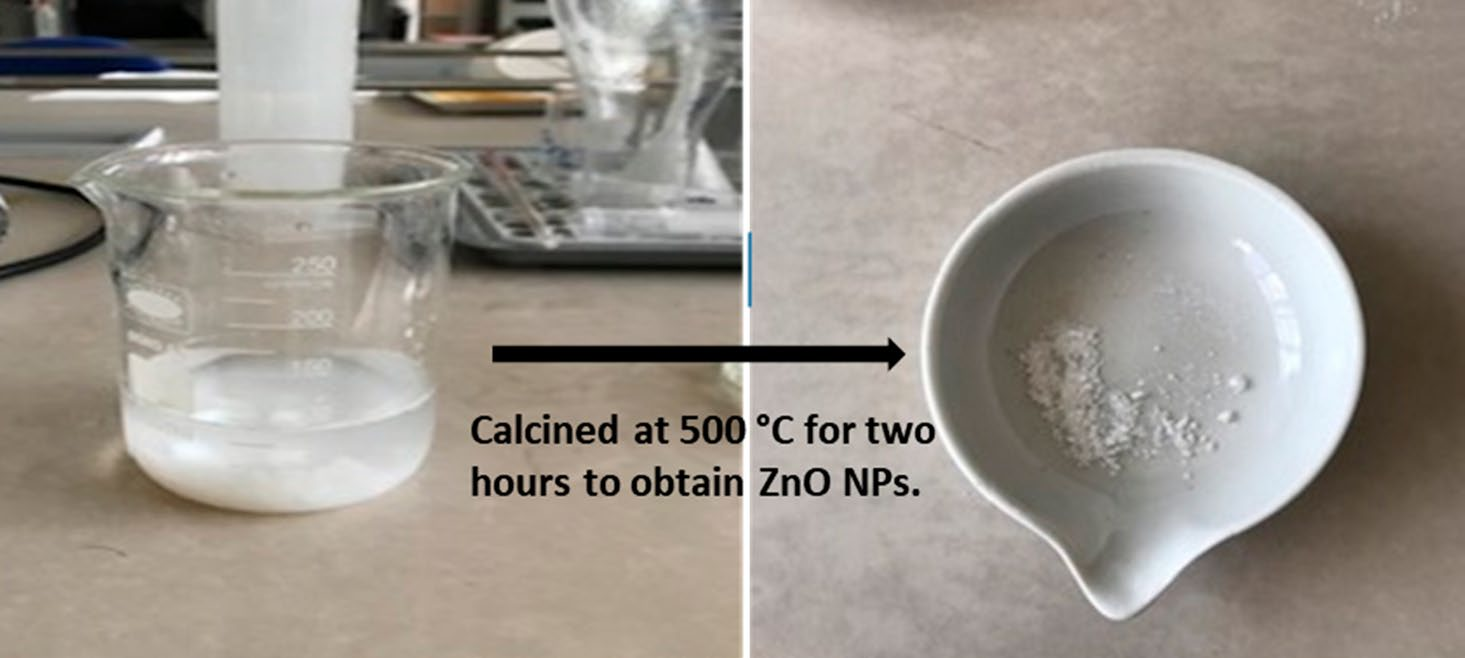
Schematic representation of ZnO nanoparticles synthesis.

ZnO nanoparticles were successfully synthesized via a precipitation method (Figure 2). Many studies have been conducted on the synthesis of ZnO nanoparticles in the literature. Baruwati et al. (2006) synthesized ZnO nanoparticles hydrothermally, whereas Talam et al. (2012) synthesized ZnO nanoparticles by precipitation using zinc nitrate (Baruwati et al. 2006; Talam et al. 2012).

### 3.3 Synthesis of ZnO/ca‐Alginate (ZnO/CA) Nanocomposite

**FIGURE 3:**
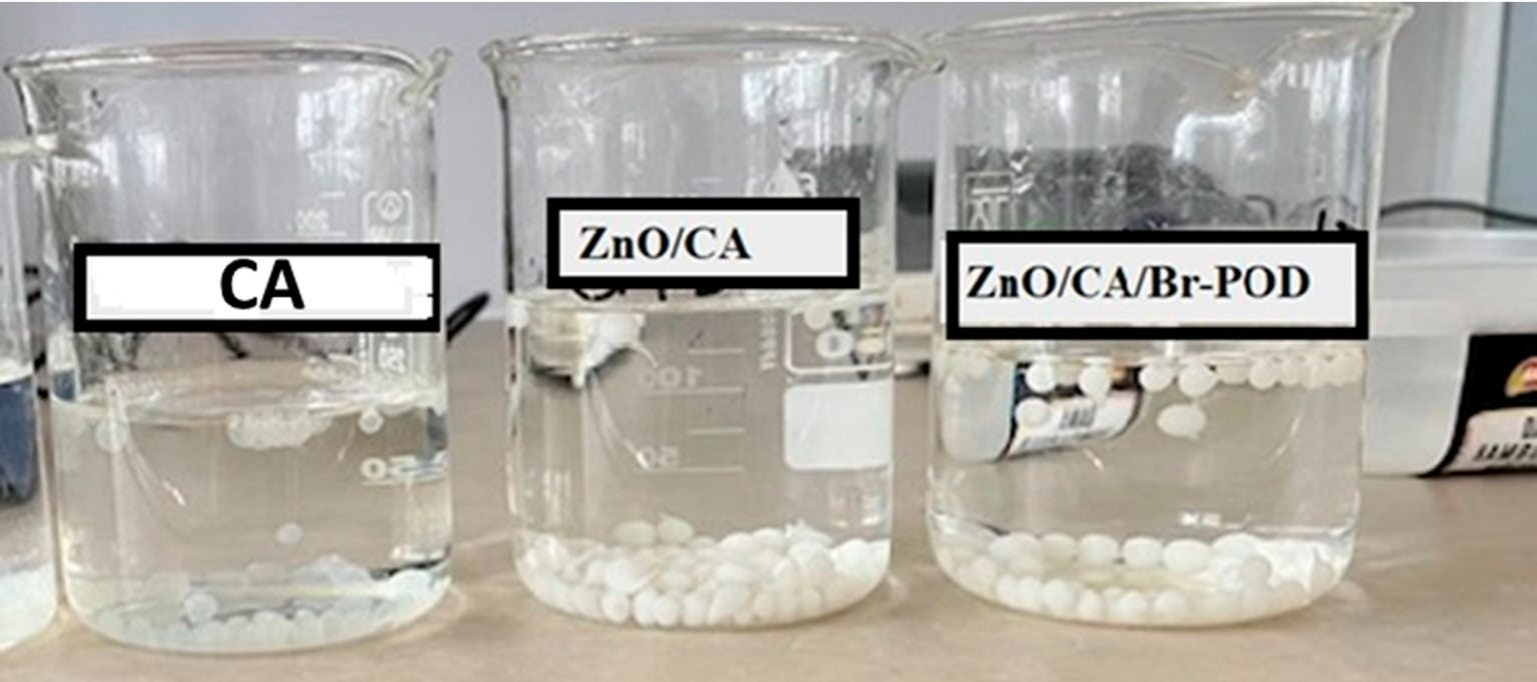
Schematic representation of the preparation of CA, ZnO/CA, and ZnO/CA/Br‐POD beads.

As shown in Figure 2, CA, ZnO/CA, and ZnO/CA/Br‐POD beads were successfully synthesized. In the synthesis process, pure CA beads were prepared, and then ZnO was added to obtain ZnO/CA beads. In the final step, ZnO/CA beads were loaded with Br‐POD, and ZnO/CA/Br‐POD composite beads were synthesized (Figure 3).

### 3.4 Optimization of Immobilization Conditions

Entrapment is commonly preferred for stabilizing enzymes and enhancing their separation and recycling capabilities. Its primary advantage is minimizing direct interactions with the enzyme during immobilization, which helps to preserve enzymatic activity. However, this benefit must be weighed against potential challenges such as enzyme partitioning or bulk transfer limitations (Imam et al. 2021). Calcium alginate entrapment is a simple, cost‐effective, efficient, and sustainable method that employs enzymes in various processes (Naveenkumar et al. 2024). In this study, Br‐POD was effectively immobilized on ZnO/CA beads. The volume and dissolution properties of the polymer used as the entrapment material are crucial for determining the successful entrapment of the enzyme. To optimize the entrapment process, key factors such as the calcium alginate and ZnO concentrations, CaCl_2_ content, and peroxidase activity within the bead microenvironment were systematically investigated (Table 2).

**TABLE 2:** Effects of the calcium alginate and ZnO concentrations, CaCl_2_ concentration, and bead size on peroxidase activity.

	Specific activity (EU/mg)
Alginate concentration % (w/v)	
1.5	6.42
2.0	6.82
2.5	7.28
3.0	7.01
ZnO (mg)	
3	5.97
6	6.14
10	6.59
CaCl_2_ concentration % (w/v)	
1.5	4.45
2.0	5.98
2.5	6.58
3.0	5.82
Bead size (mm)	
0.6	5.21
0.7	4.61
0.8	6.48

The highest enzymatic activity of ZnO/CA/Br‐POD was observed with 10 mg of ZnO and 2.5% (w/v) sodium alginate. As the gel‐forming cationic CaCl_2_ concentration increased, the immobilization efficiency improved and plateaued after 2.5% (w/v) CaCl_2_. At lower CaCl_2_ concentrations, the large pore structure of the gel led to peroxidase leakage, whereas at higher concentrations, a dense gel structure hindered substrate diffusion, resulting in no further increase in enzymatic activity (Bilal and Iqbal 2019). The optimal bead diameter was approximately 0.29 mm, which was achieved via a 0.80 × 38 mm syringe with a green tip. By optimizing these conditions, bead stiffness was increased, minimizing enzyme leakage and maximizing peroxidase activity. It is well established that intraparticle mass transfer is significantly influenced by bead size during entrapment‐based enzyme immobilization (Dalal and Gupta 2007). Smaller bead diameters generally lead to higher immobilization efficiency owing to improved mass transfer (Yildiz Dalginli and Atakisi 2023).

### 3.5 Immobilization of the Br‐POD on ZnO/CA Nanocomposite

The interaction between Br‐POD and the ZnO/CA matrix is pivotal in enhancing the biocatalytic properties of the immobilized enzyme. The carboxylate groups of alginate interact with calcium ions to form a stable gel network, while ZnO nanoparticles contribute additional functional groups (e.g., surface hydroxyls and Zn^2+^ ions) that can facilitate hydrogen bonding, electrostatic attraction, and coordination with amino acid residues on the Br‐POD enzyme. These interactions likely contribute to the observed increase in thermal stability and shift in optimal pH and temperature. In particular, Zn^2+^ ions can coordinate with imidazole (His), thiol (Cys), and carboxyl (Asp, Glu) side chains on the enzyme surface, stabilizing its tertiary structure and protecting it from denaturation under harsh conditions (Bilal et al. 2018; Shariat et al. 2018).

Moreover, the surface of ZnO nanoparticles is known to be amphoteric, allowing it to interact with both acidic and basic groups on the enzyme, which may enhance the enzyme's conformational rigidity and functional orientation (Barbosa et al. 2020). The observed shift in optimal pH for the immobilized Br‐POD may result from microenvironmental changes around the enzyme, driven by proton adsorption/desorption at the ZnO surface, altering local ionic strength and charge distribution. This modified microenvironment likely contributes to better enzyme‐substrate binding and catalysis efficiency.

The synergistic properties of ZnO and calcium alginate provide a dual‐function support system—mechanically stabilizing the matrix while also chemically interacting with the enzyme to preserve or enhance its function under operational conditions.

In this study, Br‐POD was immobilized via the ZnO/CA entrapment method to increase its catalytic activity and thermostability. As shown in Table 3, the specific activity of the immobilized Br‐POD was 5.97 EU/mL, the protein loading was 0.230 mg/mL, and the immobilization efficiency was 90.6%. Notably, the immobilized enzyme retained 80.8% of its initial activity.

**TABLE 3:** Protein amount, specific activity, and immobilization efficiency results of ZnO/CA prepared via the entrapment method.

	Br‐POD enzyme	ZnO/CA/Br‐POD
Activity (EU/mL)	1.701	1.374
Protein (mg/mL)	0.258	0.230
Specific activity (EU/mg)	6.59	5.97
İmmobilization effectiveness (%)	—	90.6

Similar studies have reported high encapsulation efficiencies for HRP immobilized on calcium alginate‐gelatin hybrid hydrogel beads (91.25%). The specific activities of free HRP and immobilized HRP were determined to be 4.416 and 6.708 μmol min^−1^ mg protein^−1^, respectively (Yildiz Dalginli et al. 2024). Furthermore, peroxidase immobilized on a silica SBA‐15/albumin hybrid and coconut fiber through covalent bonding exhibited encapsulation efficiencies of 89.99% and 77.74%, respectively (Barbosa et al. 2020).

### 3.6 Surface Chemical Structure Analysis

#### 3.6.1 FT‐IR Spectra of the Prepared Films

**FIGURE 4:**
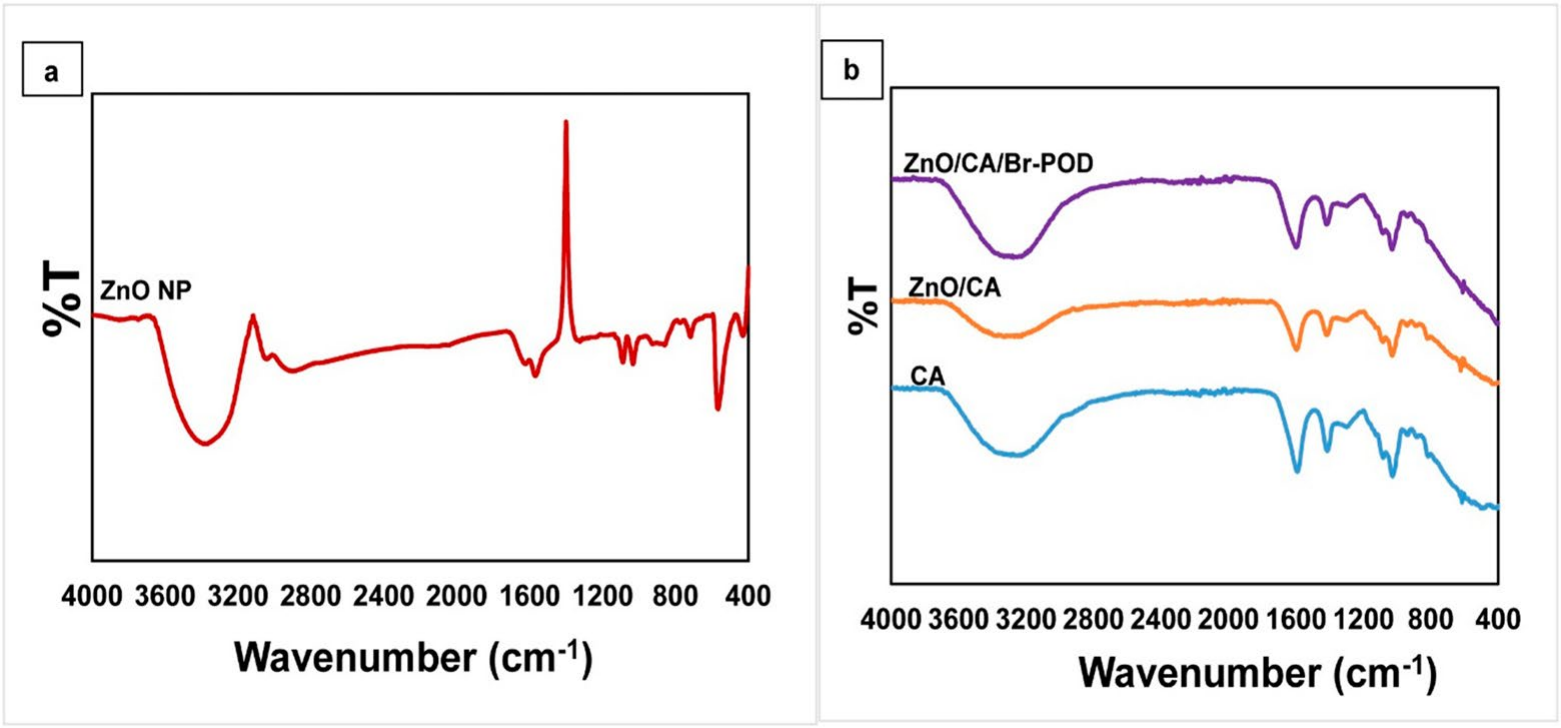
(a) FT‐IR spectrum of ZnO nanoparticles showing characteristic Zn–O stretching vibrations. (b) FT‐IR spectra of CA, ZnO/CA, and ZnO/CA/Br‐POD beads confirming successful incorporation of components. (a) shows that the FT‐IR spectrum of the ZnO nanoparticles exhibited characteristic ZnO stretching vibrations in the 400–800 cm^−1^ range. The peak at 3446 cm^−1^ is attributed to the O‐H stretching vibration of the water adsorbed on the metal oxide surface.

FT‐IR‐ATR spectroscopy was used to investigate the surface chemistry and functional groups of the ZnO NPs and the CA, ZnO/CA, and ZnO/CA/Br‐POD beads. The resulting spectra are shown in Figure 4a,b.

Figure 4b presents the characteristic IR bands of alginate in CA beads, particularly in the O—H stretching (∼3200–3400 cm^−1^), C=O stretching (∼1600 cm^−1^), and C—O—C stretching (∼1030–1100 cm^−1^) regions. The O—H and C=O groups of alginate indicate its hydrophilic nature and its ionic cross‐linking propensity (Weber et al. 2023). Upon the addition of ZnO to form ZnO/CA beads, a new peak emerged in the 400–500 cm^−1^ region, indicating the presence of Zn‐O bonds and confirming the successful integration of ZnO nanoparticles into the CA matrix. Slight shifts in the characteristic alginate bands were also observed, reflecting interactions between ZnO and the alginate functional groups (Batool et al. 2024).

In the ZnO/CA/Br‐POD beads, additional spectral changes were noted, primarily in the O‐H and N‐H stretching regions (∼3200–3500 cm^−1^), indicative of the presence of enzyme molecules. Furthermore, the prominent amido I and amido II bands of proteins (∼1650 cm^−1^ and 1550 cm^−1^, respectively) confirmed the successful immobilization of Br‐POD onto the ZnO/CA matrix. These spectral alterations support the formation of bonds and surface interactions between the enzyme and the ZnO/CA matrix (Weber et al. 2023).

FT‐IR spectroscopy effectively identified the functional groups on the surfaces of CA, ZnO/CA, and ZnO/CA/Br‐POD beads. The observed spectral changes upon adding ZnO and Br‐POD confirm the successful integration of each component into the structure and the resulting modifications in the functional properties of the material.

#### 3.6.2 Morphological Studies

##### 3.6.2.1 EDS Analysis and SEM Images of CA, ZnO/CA, and ZnO/CA/Br‐POD Beads

**FIGURE 5:**
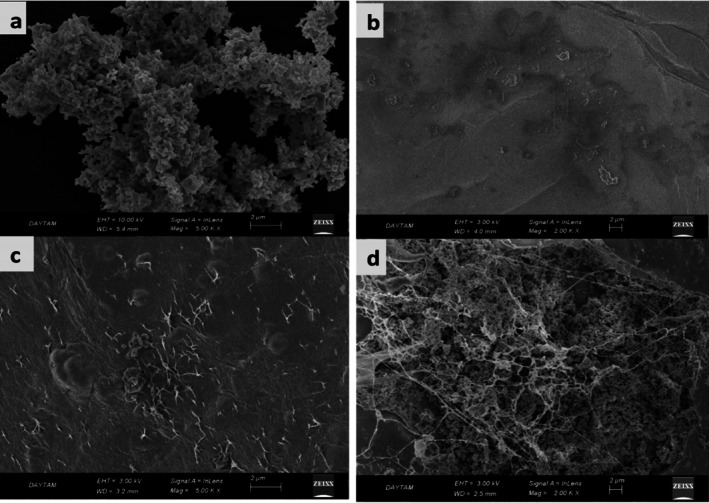
SEM micrographs showing surface morphology changes during the development of composite beads: (a) ZnO nanoparticles showing agglomerated particles, (b) Smooth‐surfaced calcium alginate (CA) beads, (c) ZnO/CA beads with visible ZnO distribution, and (d) ZnO/CA/Br‐POD beads showing enzyme immobilization effects. (a) shows an SEM image of pure ZnO powder, where generally spherical or slightly oval‐shaped nanoparticles are observed. The particles tend to cluster and agglomerate, forming irregular structures. Despite this, the particles appear relatively homogeneous in size and maintain a predominantly spherical or slightly ellipsoidal morphology. Agglomeration, characterized by particle clustering, is evident in certain regions. This morphology suggests that ZnO nanoparticles possess a high surface area, potentially contributing to their high reactivity (Zyoud et al. 2023). The synthesis conditions, such as temperature, pH, and precursors, as well as the methods employed (e.g., chemical precipitation, sol–gel), significantly influence the morphology of the ZnO nanoparticles. Various ZnO nanoparticle morphologies, including rod‐like, wire‐like, flower‐like, and spherical forms, have been reported in the literature. The morphology observed in this image aligns with findings reported in the literature (Muhammad et al. 2019).

**FIGURE 6:**
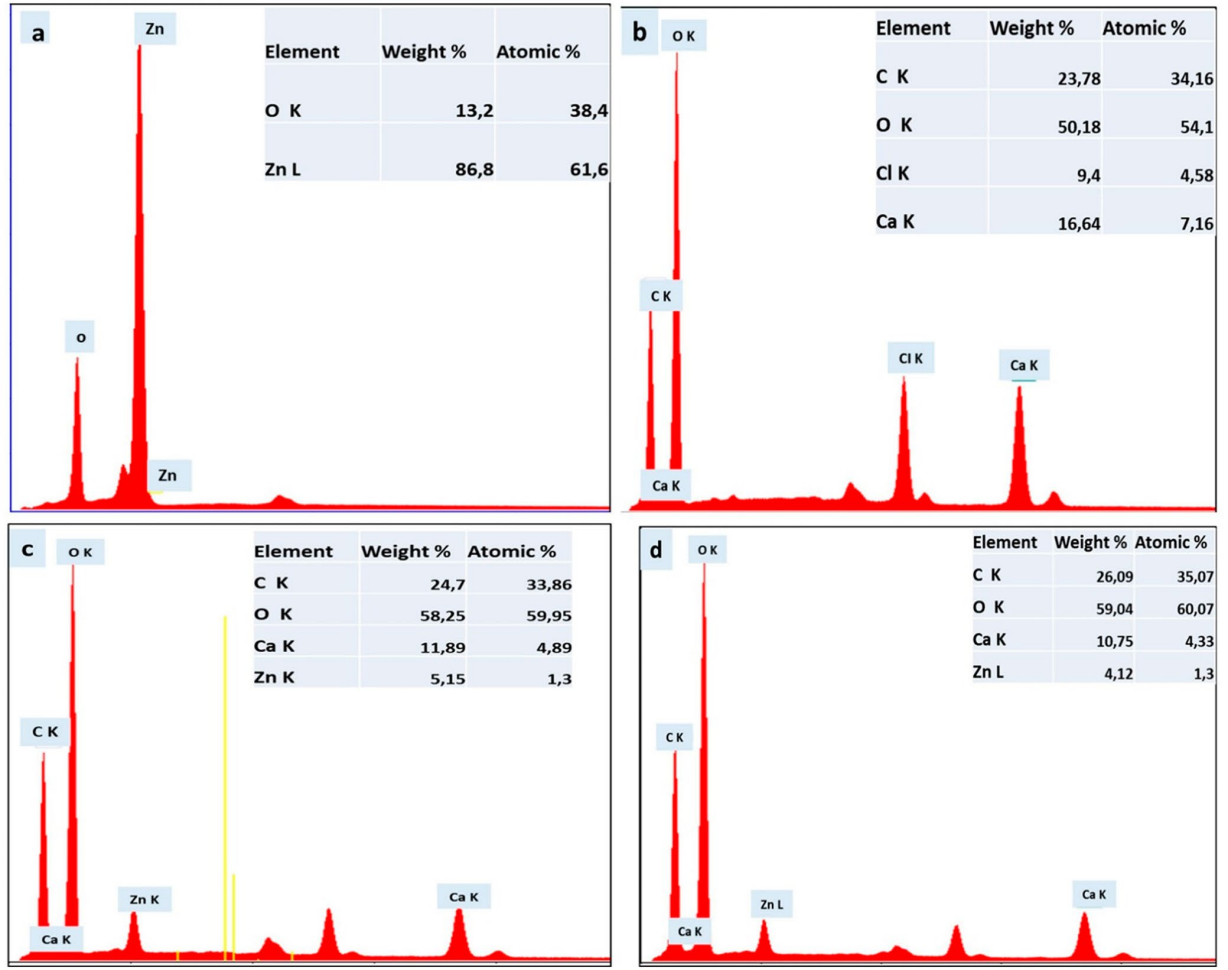
EDS spectra of (a) ZnO nanoparticles, (b) CA beads, (c) ZnO/CA beads, and (d) ZnO/CA/Br‐POD beads confirming elemental composition.

Figure 5 presents SEM micrographs of all the materials, whereas Figure 6 displays the corresponding energy‐dispersive X‐ray spectroscopy (EDS) images.

Figure 5b shows the SEM image of CA beads, which exhibit a smooth structure without visible pores, indicating the film's homogeneity and structural integrity.

Figure 5c shows an SEM image of the ZnO/CA beads. The ZnO particles are observed to be uniformly distributed within the matrix, forming visible accumulations that appear as white dots. This distribution pattern becomes increasingly dense as the nanoparticle loading increases (Vahidhabanu et al. 2017).

Figure 5d presents an SEM image of the ZnO/CA/Br‐POD beads. Before immobilization, the surface of the beads exhibited a homogeneous pattern with unconnected pores. Following immobilization, the surface became covered with larger particles, which appeared fuller and denser. This change suggested that the Br‐POD enzyme either adsorbed onto the surface of the ZnO/CA composite or penetrated the pores, significantly altering the surface morphology.

Energy‐dispersive X‐ray spectroscopy (EDS) analysis was conducted to confirm the incorporation of ZnO into the composite and to determine the elemental composition of the various materials. Figure 6a–d presents the EDS spectra of the analyzed samples.

The EDS spectrum of the ZnO/CA beads (Figure 6c) indicates the presence of zinc (Zn), calcium (Ca), carbon (C), and oxygen (O) elements. The detection of Zn confirmed the successful incorporation of ZnO nanoparticles into the CA matrix, resulting in the desired hybrid material. To validate the successful immobilization of Br‐POD onto the ZnO/CA beads, a comparative analysis of the carbon and oxygen atomic weights was performed between the immobilized sodium alginate beads (Figure 6d) and the enzyme‐free alginate beads (Figure 6c). The results revealed a significant increase in the carbon and oxygen contents of the immobilized microspheres compared with those of blank microspheres. Specifically, the immobilized ZnO/CA/Br‐POD sample presented a carbon content of 35.07% and an oxygen content of 60.07%.

#### 3.6.3 Crystal Structure Analysis

##### 3.6.3.1 XRD Analysis of ZnO np, ZnO/CA, and ZnO/CA/Br‐POD Beads

**FIGURE 7:**
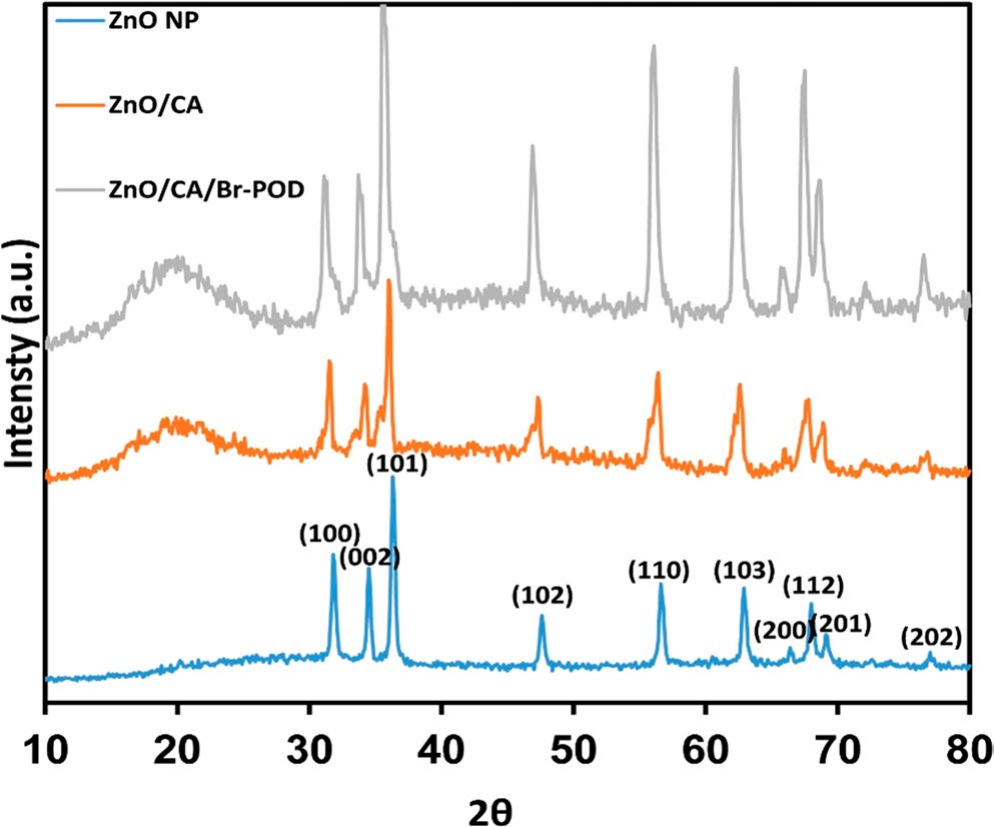
XRD analysis of ZnO Np, ZnO/CA, and ZnO/CA/Br‐POD beads.

The XRD pattern was analyzed over diffraction angles ranging from 10° to 80°, and the corresponding pattern is presented in Figure 7.

XRD analysis confirmed the crystalline phases of the ZnO NP, ZnO/CA, and ZnO/CA/Br‐POD samples. The diffraction peaks for these materials at 2θ values of 31.8355°, 34.5207°, 36.2871°, 47.5808°, 56.6487°, 62.8917°, 66.4115°, 67.9331°, 69.1842°, and 77.0106° correspond to the (100), (002), (101), (102), (110), (103), (200), (112), (201), and (202) crystal planes respectively. These findings align with those of the standard crystal plane data (JCPDS card No. 01–007‐2551) (Muhammad et al. 2019). Furthermore, the results are consistent with similar studies reported in the literature (Naveenkumar et al. 2024).

### 3.7 Biochemical Characterization

#### 3.7.1 Effects of pH and Temperature on the Activity and Stability of Free and Immobilized Peroxidase

**FIGURE 8:**
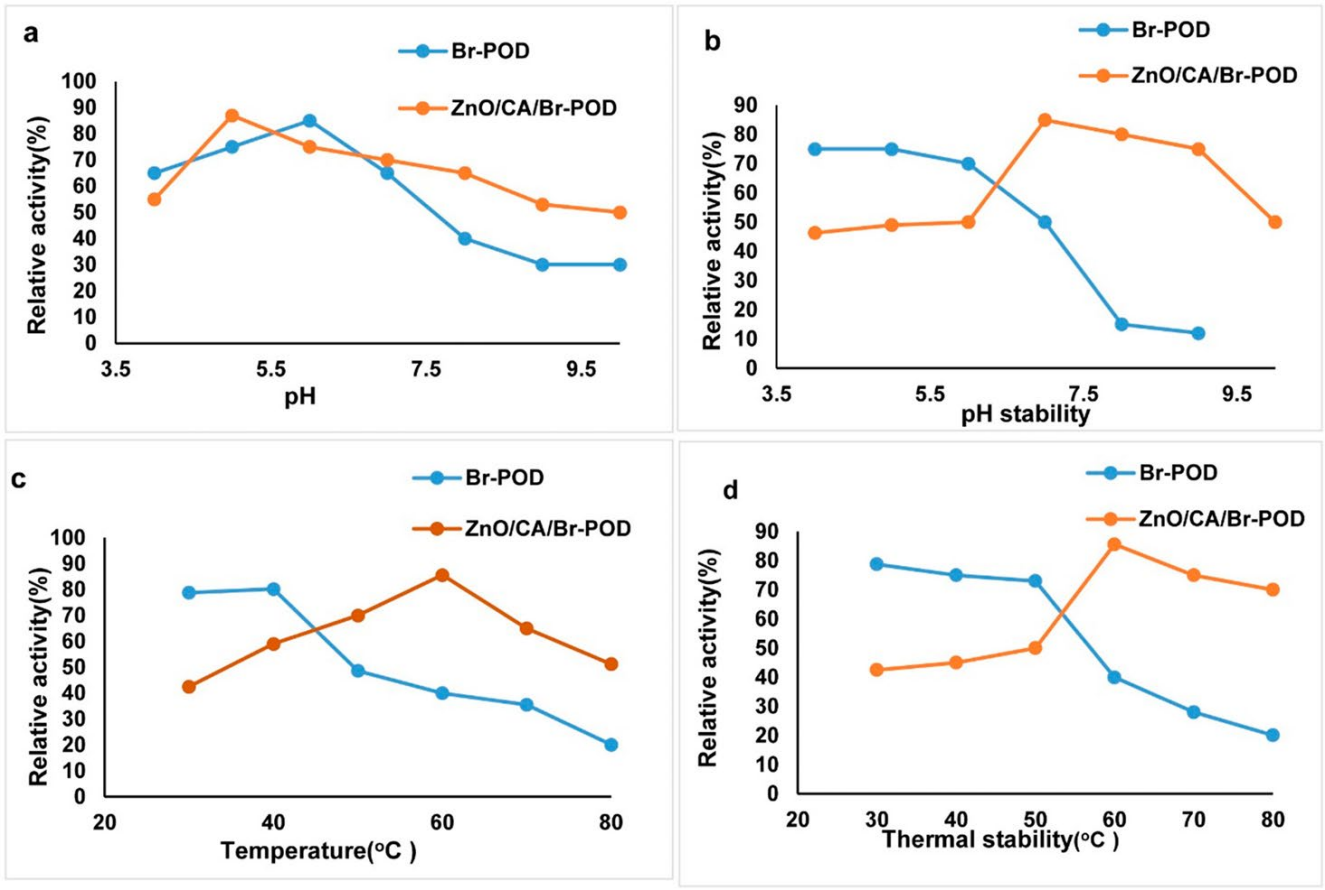
Effect of (a) pH, (b) pH stability, (c) temperature, and (d) thermal stability on the activity of free and immobilized Br‐POD.

pH and temperature parameters significantly influence the activity of various enzymes and their applications in different industrial sectors. This study measured peroxidase activity over a pH range of 4–10 for 30 min at 37°C. The optimum pH values for free Br‐POD and ZnO/CA/Br‐POD were 6 and 5, respectively (Figure 8a). The observed difference of approximately 1 pH unit between free Br‐POD and ZnO/CA/Br‐POD may be attributed to changes in the surface properties of the carrier material, which was modified to include additional amino groups. This modification enables the carrier to absorb H^+^ ions from the surrounding solution, resulting in a localized decrease in pH around the enzyme. The increased concentration of amino groups on the modified carrier facilitates proton absorption, altering the enzyme's microenvironment and optimum pH conditions (Bilal et al. 2018). Additionally, the amphoteric nature of ZnO creates a buffered microenvironment near the enzyme, facilitating enhanced proton exchange and maintaining the optimal ionic strength for catalytic activity (Barbosa et al. 2020). This microenvironmental modulation can explain the shift in both optimal pH and temperature values upon immobilization. Similar effects have been reported with ZnO/chitosan matrices (Bilal et al. 2018). A literature review revealed that peroxidases from different sources present optimum pH values within similar ranges. For example, peroxidase has been immobilized on a green nanoadsorbent through covalent bonding, where chitosan was cross‐linked to graphene oxide via electrostatic interactions (Alatawi et al. 2020).

To assess pH stability, free Br‐POD and ZnO/CA/Br‐POD were incubated for 1 h across a pH range of 4–10 under their respective optimum temperatures (Figure 8b). The results indicated that the free enzyme was more stable than the immobilized enzyme under acidic conditions (pH 4–6). However, the immobilized enzyme retained approximately half of its activity under highly alkaline conditions and demonstrated better stability than the free enzyme.

The optimum temperatures for the free enzyme and ZnO/CA/Br‐POD were tested within the 30°C–80°C range and determined to be 40°C and 60°C, respectively. The free enzyme lost approximately 38.8% of its activity at 50°C and 60°C, whereas the immobilized enzyme retained almost complete activity under the same conditions (Figure 8c). These findings indicate that the free enzyme exhibited greater activity at lower temperatures, whereas the immobilized enzyme performed better at elevated temperatures. At high temperatures such as 60°C, 70°C, and 80°C, the immobilized enzyme retained approximately 85.6%, 65%, and 51.2% relative activity, respectively, whereas the free enzyme exhibited approximately 40%, 35.5%, and 20.1% activity, respectively, under the same conditions. Compared with their free counterparts, immobilized enzymes are generally characterized by higher optimum temperatures than their free counterparts. For instance, the optimum temperature of a peroxidase‐concanavalin complex immobilized on calcium alginate pectin gel was reported to be 60°C. Similarly, the optimum temperature of peroxidase immobilization on iron oxide nanoparticles increases from 35°C to 50°C after immobilization (Kalsoom et al. 2023).

In the present study, the optimum temperature increased from 40°C to 60°C upon immobilization, making the enzyme more suitable for industrial applications requiring high temperatures. Additionally, the thermal stability of free Br‐POD and ZnO/CA/Br‐POD was evaluated in the 30°C–80°C temperature range over a 1‐h incubation period (Figure 8d). The free enzyme retained nearly all of its activity at low temperatures but lost most of its activity at high temperatures. In contrast, the immobilized enzyme demonstrated enhanced thermal stability, retaining approximately 75% and 70% of its relative activity at 70°C and 80°C, respectively, after 1 h of incubation.

This increase in thermal stability can be attributed to changes in molecular interactions and reduced flexibility between the biocatalyst and the support medium following immobilization (Hermanová et al. 2015).

#### 3.7.2 Reusability and Storage Stability of Free and Immobilized Enzymes

**FIGURE 9:**
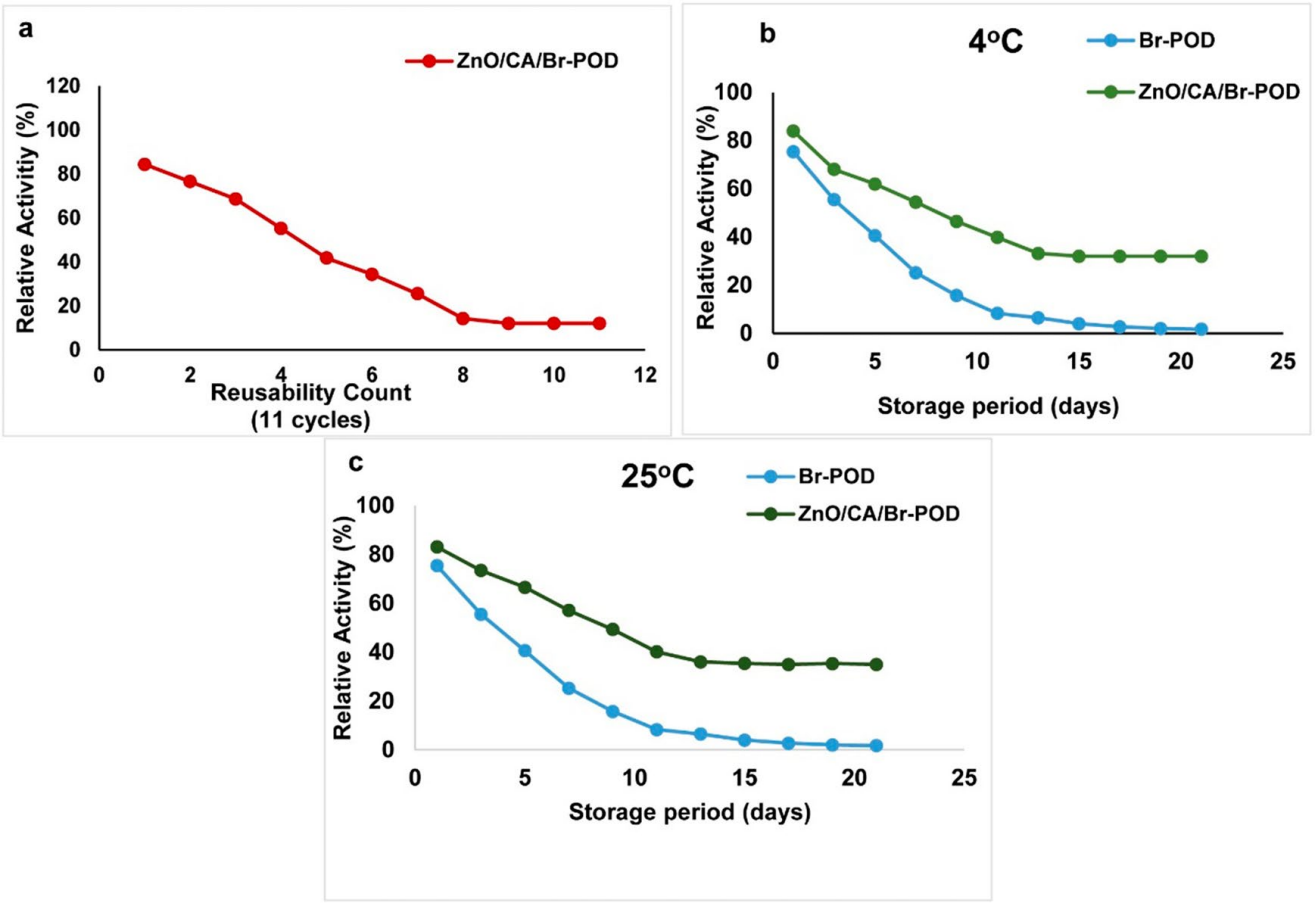
(a) Reusability of immobilized Br‐POD over multiple cycles. (b) Storage stability at 4°C. (c) Storage stability at 25°C.

Low reusability, low stability, and high cost are key factors limiting the widespread use of enzymes in industrial applications (Bilal and Iqbal 2019). The study of enzyme reusability holds significant economic importance, particularly for industrial applications. This study assessed the reusability of ZnO/CA/Br‐POD over 12 cycles under optimum conditions. The enzyme retained approximately 76.5%, 68.6%, 55.2%, and 41.7% of its relative activity during the 2nd, 3rd, 4th, and 5th cycles, respectively. However, after the 11th cycle, the ZnO/CA/Br‐POD lost its activity (Figure 9a). This loss of activity is likely due to the application and washing during. Beyond mechanical degradation, repeated use may also cause enzyme leaching or conformational changes due to cumulative exposure to substrates and shear stress. However, the high retention observed in the first few cycles supports the physical and chemical stability conferred by the ZnO/CA matrix, as also demonstrated by Liu et al. (2023).

Similarly, Urrea et al. (2021) reported that horseradish peroxidase (HRP) immobilized on calcium alginate beads retained its activity after four hydrolysis cycles (Urrea et al. 2021). In another study, Abd Rahim et al. (2013) reported that the covalent immobilization of HRP onto enzymes encapsulated in calcium alginate‐clay beads resulted in 51.77% retention of activity after seven hydrolysis cycles (Abd Rahim et al. 2013).

The stability of free and immobilized LPO was monitored during storage at 4°C and 25°C for 21 days by measuring their daily activities. ZnO/CA/Br‐POD retained 22.3% and 23.7% of its initial activity after 21 days of storage at 4°C and 25°C, respectively. In contrast, free Br‐POD lost its activity entirely after 7 days (Figure 9b,c).

Numerous studies have demonstrated that immobilization can significantly increase the storage stability of enzymes. For example, HRP immobilized on glutaraldehyde‐functionalized rice straw biochar exhibited greater activity than the free enzyme did, retaining approximately 90% of its initial activity after 30 days of storage at room temperature (Liu et al. 2023). Similarly, immobilized HRP retained 59% of its initial activity after 2 months and 28.8% after 3 months, whereas free HRP lost 92.7% of its activity after 2 months and was inactivated entirely after 75 days of storage (Abdulaal et al. 2020).

### 3.8 Antioxidant Assays

#### 3.8.1 CA and ZnO/CA/Br‐POD Beads DPPH and ABTS Radical Scavenging Assay

Free radicals and reactive oxygen species (ROS) can induce lipid peroxidation and protein denaturation in food, leading to spoilage and a reduced shelf life. It has been reported that incorporating antioxidant substances into food packaging films or beads can significantly enhance their antioxidant performance (Ge et al. 2022). This study incorporated ZnO nanoparticles (ZnO NPs) and Br‐POD enzymes into calcium alginate (CA) beads to confer antioxidant properties.

**FIGURE 10:**
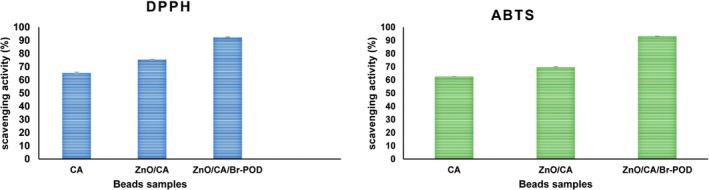
DPPH and ABTS radical scavenging activities of CA, ZnO/CA, and ZnO/CA/Br‐POD beads at 25 mg/mL. Data are expressed as mean ± SD (*n* = 3). Statistical significance was determined using the two‐way ANOVA (*p* < 0.05).

The antioxidant potential of the beads was evaluated using DPPH and ABTS radical scavenging assays (Figure 10). As shown in the results, CA beads demonstrated scavenging rates of 65.33% ± 0.577% (DPPH) and 62.4% ± 0.100% (ABTS), which increased significantly after ZnO incorporation (*p* < 0.05) to 75.36% ± 0.152% and 69.9% ± 0.605%, respectively. The ZnO/CA/Br‐POD beads exhibited the highest activity, with DPPH and ABTS scavenging rates of 92.36% ± 0.404% and 93.4% ± 0.057%, respectively.

The superior antioxidant effect of the Br‐POD‐immobilized beads is primarily due to the Br‐POD enzyme's catalytic ability to convert hydrogen peroxide into water, thereby neutralizing reactive oxygen species (ROS). This mechanism mirrors that of other antioxidant enzymes used in active packaging systems (e.g., HRP, LPO) as reported in similar studies (Behbahani et al. 2024; Ton‐That et al. 2025; Zanganeh et al. 2021). Moreover, the peroxidase enzyme (Br‐POD) is capable of reacting not only with H_2_O_2_ but also with a wide variety of phenolic and nonphenolic substrates. This versatile catalytic mechanism contributes to the reduction of oxidative stress in both food matrices and biological systems (Alotaibi et al. 2025; Gulcin 2025).

Similar to our observations, it has been demonstrated that enzyme‐functionalized coatings significantly enhance DPPH and ABTS scavenging capacities in composite films. These findings are consistent with earlier reports on the incorporation of antioxidant enzymes and natural extracts into films to enhance the oxidative stability of food matrices, supporting the use of biocatalyst‐embedded packaging materials to preserve freshness and quality (Chuysinuan et al. 2018; Mustafa and Andreescu 2020; Xie et al. 2025).

### 3.9 Agar Well Diffusion Assays of Prepared Films

Microbial growth is a critical factor influencing the safety of food products. It can lead to food spoilage and pose significant health risks through foodborne illnesses. To mitigate microbial contamination, antimicrobial substances are often incorporated into packaging materials to inhibit microbial growth (Sani et al. 2019).

**FIGURE 11:**
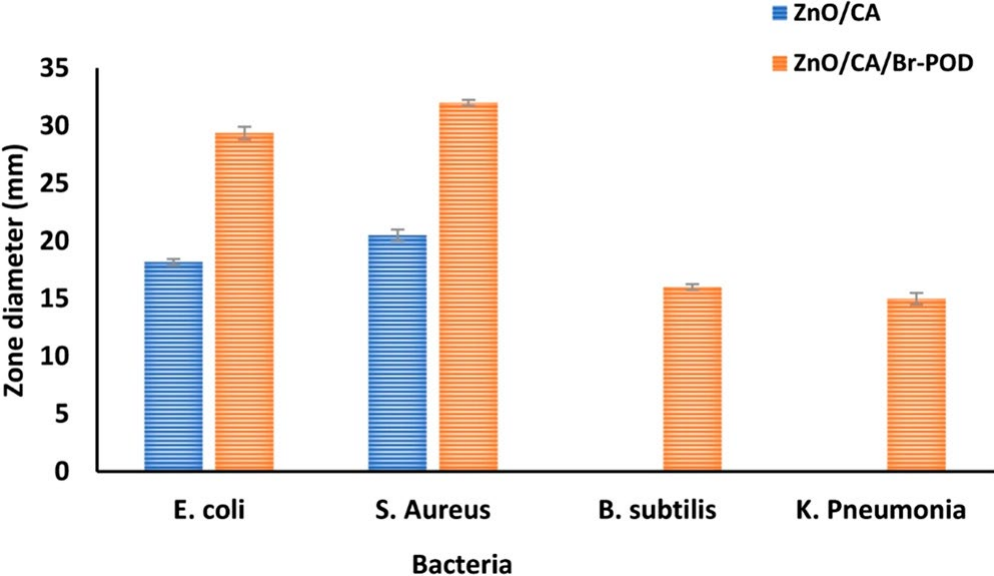
Antibacterial activity of CA, ZnO/CA, and ZnO/CA/Br‐POD beads against selected bacterial strains based on inhibition zones.

The antibacterial activity of CA, ZnO/CA, and ZnO/CA/Br‐POD beads was evaluated by measuring the inhibition zone diameters using the agar well diffusion method (Table 4, Figure 11). Statistical analysis using two‐way ANOVA revealed significant differences among the tested groups (*p* < 0.05), confirming the superior efficacy of ZnO/CA/Br‐POD beads compared to CA and ZnO/CA alone.

**TABLE 4:** Antibacterial properties of alginate beads by the agar well method.

Bacteria	Beads^a^		Positive control^b^
	ZnO/CA	ZnO/CA/Br‐POD	CA
GR (+) bacteria			
B1	—	16.12 ± 0.25	—
B2	20	32.15 ± 0.25	—
GR (−) bacterica			
B3		29.36 ± 0.55	—
B4	—	15.31 ± 0.50	—

*Note:* The inhibitory zone (mm) diameter for different concentrations (25 mg. mL^−1^).

Abbreviations: −, inhibition zone not formed; B1, *Bacillus subtilis* ; B2, *Staphylococcus aureus* ; B3, *Escherichia coli* ; B4, *Klebsiella pneumonia*; GR−, GR negative; GR+, GR positive a) ZnO/CA, ZnO/CA/Br‐POD, b) CA.

The antibacterial activities of ZnO/CA and ZnO/CA/Br‐POD beads at a concentration of 25 mg/mL were evaluated against gram‐positive bacteria (*Streptococcus pyogenes ATCC 19615* and *Staphylococcus aureus ATCC 25923*) and gram‐negative bacteria (*Escherichia coli O157* and *Bacillus cereus ATCC 14579*). The results, presented in Figure 10 and Table 4, are reported in millimeters (mm). The CA beads showed no activity against any bacterial strains. In contrast, ZnO/CA beads exhibited moderate activity due to the antimicrobial properties of Zn^2+^ ions, which are known to disrupt microbial membranes by interacting with proteins containing imidazole, thiol, and carboxyl groups (Thomas et al. 2018). This antimicrobial activity is generally more pronounced against Gram‐positive bacteria due to their thicker peptidoglycan layers and less complex outer membrane structure, which facilitates better penetration and membrane disruption by reactive oxygen species (ROS) generated by Br‐POD activity (Deshmukh and Gaikwad 2024).

The incorporation of Br‐POD further enhanced the antibacterial activity, with inhibition zones reaching 32.15 ± 0.25 mm against *Staphylococcus aureus* and 29.36 ± 0.55 mm against *Escherichia coli* . This enhanced effect is attributed to the oxidative stress generated by the peroxidase‐mediated conversion of H_2_O_2_ into reactive radicals, which compromise bacterial membrane integrity and lead to cell death. (Deshmukh and Gaikwad 2024; Noshad et al. 2022).

These results are consistent with previous studies in which peroxidase enzymes immobilized on nanocomposites demonstrated strong bactericidal effects via oxidative stress mechanisms and direct enzyme–cell wall interactions. Our findings are also in line with previous reports using enzyme‐loaded edible films with potent antimicrobial functionality, particularly against Gram‐negative pathogens (Alizadeh Behbahani and Imani Fooladi 2018; Thomas et al. 2018).

Several recent studies have investigated the use of natural enzymes, essential oils, plant extracts, nanoparticles, or composite films for active food packaging and antimicrobial protection. For example, Tanavar et al. (2021) incorporated essential oils and natural antimicrobials into biodegradable films, observing similar improvements in microbial suppression and shelf‐life extension. Likewise, Alizadeh Behbahani and Imani Fooladi (2018) used *Ocimum basilicum* seed mucilage edible films to enhance the antimicrobial functionality of food packaging systems.

### 3.10 Testing of Packaging Applications and Biodegradation

During storage, weight loss in fruits and vegetables occurs due to water evaporation and respiratory cycles, during which glucose is converted into carbon dioxide. This process reduces sensory quality, softens the product, and produces less desirable results. Consequently, weight loss is one of the most critical factors influencing the likelihood of postharvest spoilage in fruits and vegetables. The *Agaricus bisporus* (JE Lange) Imbach used in this study, commonly known as the button mushroom, is an edible fungal species belonging to the family Agaricaceae. It comprises three subspecies with distinct reproductive strategies: *A. bisporus var. bisporus, A. bisporus var. burnettii, and A. bisporus var. Eurotetrasporus* (Chung Ill Min et al. 2018). For mushrooms, weight loss is attributed primarily to metabolic reactions associated with the ripening process (Muley et al. 2022). This deterioration is further accelerated by the movement of water from the mushroom tissue into the surrounding environment (Shankar et al. 2021). Appropriate packaging can be an effective barrier to prevent water loss while limiting the interaction of oxygen and carbon dioxide with the product. Doing so slows respiratory and metabolic activities, thereby preserving the quality of the mushrooms.

**FIGURE 12:**
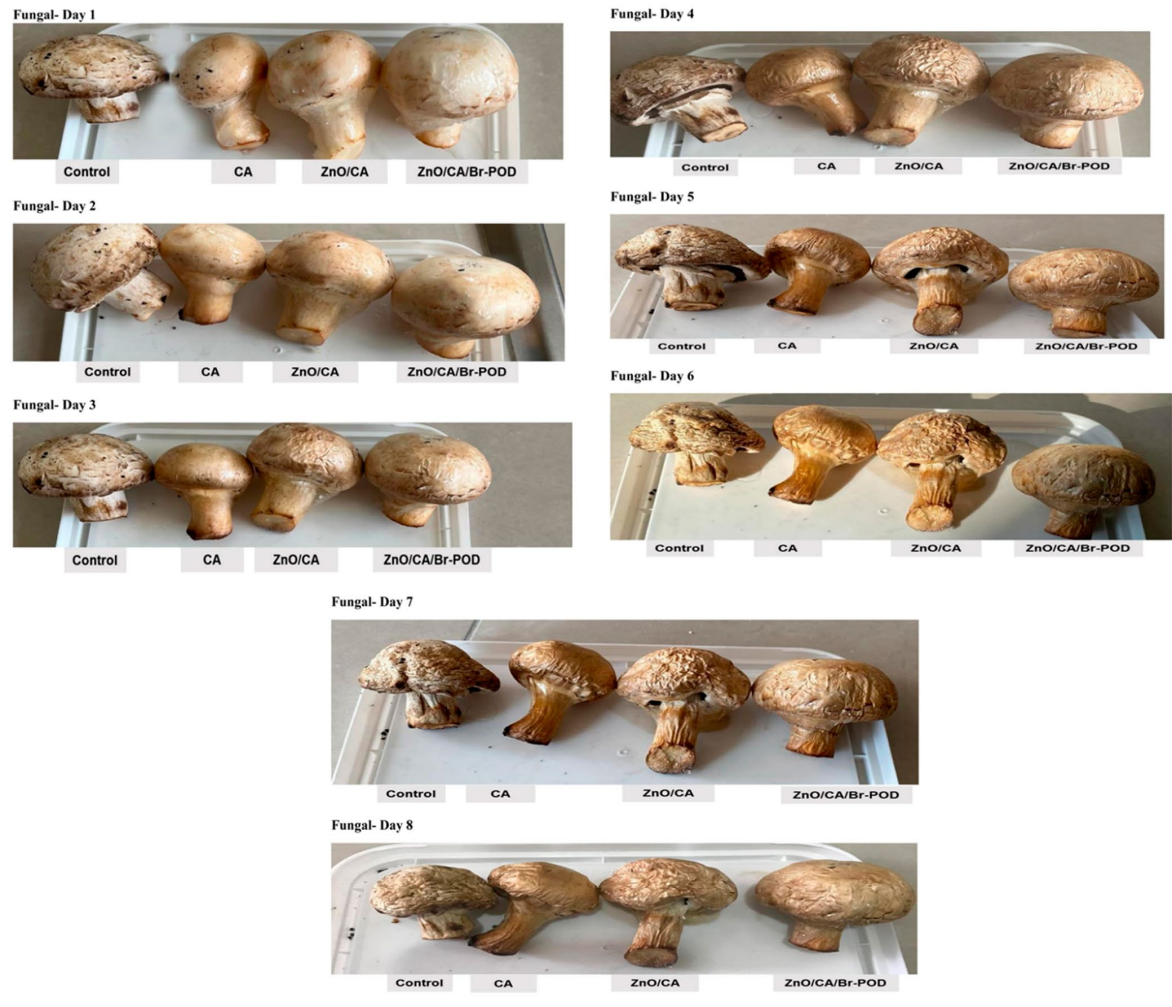
Visual appearance of mushrooms after storage under different packaging conditions over 8 days.

**FIGURE 13:**
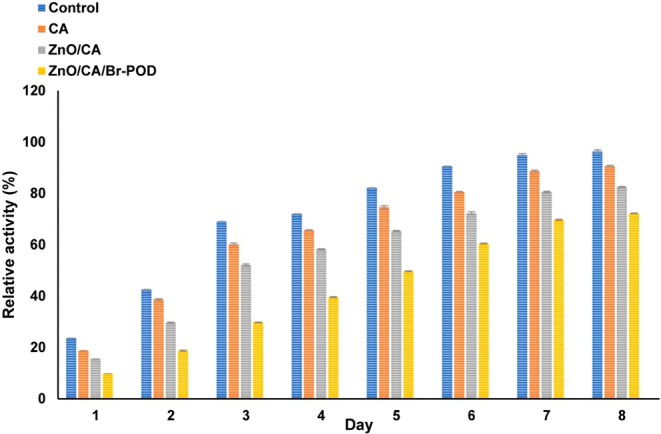
Percentage weight loss (%) of *Agaricus bisporus* mushrooms over an 8 under different packaging conditions (CA, ZnO/CA, and ZnO/CA/Br‐POD coatings). Data indicate the preservation effect of bioactive beads compared to the control group.

Figures 12 and 13 present the sensory evaluation results of the mushrooms and their weight loss after an 8‐day storage period. Significant differences were observed among the prepared alginate bead samples. As expected, the weight loss of the wrapped mushrooms decreased across all the groups throughout the storage period. Notably, vegetable samples wrapped with ZnO/CA/Br‐POD beads exhibited more effective protection against spoilage (Figure 12). In addition to the physical barrier function of the coating, the biochemical action of Br‐POD contributed to the preservation effect by catalyzing the conversion of endogenous H_2_O_2_ into water, limiting oxidative stress in the mushroom tissues. This is crucial, as oxidative stress during postharvest storage is a significant cause of browning, softening, and senescence in mushrooms (Hafeez et al. 2024; Saleem et al. 2021). This enhanced protection can be attributed to the interaction between the negatively charged phospholipids in the fungal plasma membrane and the positively charged amino chains of the Br‐POD enzyme in the ZnO/CA/Br‐POD beads. This interaction disrupts fungal cell permeability, leading to the loss of cellular components (Hafeez et al. 2024).

Postharvest storage conditions often trigger the formation of reactive oxygen species (ROS), which can cause oxidative damage in fruits and vegetables (Saleem et al. 2021). Antioxidant enzymes, such as superoxide dismutase (SOD), peroxidase (POD), and catalase (CAT), play crucial roles in scavenging ROS and protecting tissues from oxidative damage (Salih et al. 2024).

As illustrated in Figure 13, the control group experienced a 69.00% ± 0.099% weight loss by the third day, whereas the samples wrapped with CA and ZnO/CA beads exhibited weight losses of 60.13% ± 0.152% and 52.13% ± 0.251%, respectively. Notably, the samples wrapped with ZnO/CA/Br‐POD beads demonstrated superior performance, losing only 39.56% ± 0.152% of their weight by the fourth day. By the end of the eighth day, the control group had lost 96.46% ± 0.305% of its initial weight (*p* < 0.05). In contrast, the mushrooms coated with ZnO/CA/Br‐POD beads retained their integrity better, losing only 72.20% ± 0.56% of their weight (Figure 13) Moreover, the integration of ROS‐scavenging enzymes into the packaging matrix provides a biological defense mechanism that complements the physical barrier, resulting in a synergistic enhancement of product preservation (Mosallaie et al. 2024; Salih et al. 2024).

The application of ZnO/CA/Br‐POD beads in extending the shelf life of Agaricus bisporus mushrooms demonstrated their practical functionality (Figure 12). The immobilized beads reduced weight loss, browning, and microbial spoilage over the storage period. These effects are likely due to the combined antimicrobial and antioxidative actions of the system.

This is supported by Mosallaie et al. (2024), who showed that bioactive edible coatings enriched with antimicrobial compounds significantly improved the postharvest quality and storability of fresh mushrooms. Moreover, the peroxidase‐like catalytic mechanism, which contributes to reactive oxygen species scavenging, was similarly observed by Salehi et al. (2024) in the degradation of chemical pollutants using immobilized biocatalysts, highlighting the versatile applications of such enzyme systems.

## 4 Conclusions

This study demonstrates the successful development of a novel biocatalytic system by immobilizing black radish peroxidase (Br‐POD) onto ZnO‐loaded calcium alginate (ZnO/CA) beads, resulting in significantly enhanced enzyme stability, thermal tolerance, and pH tolerance, as well as improved antioxidant and antimicrobial activities. The multifunctional nature of the ZnO/CA/Br‐POD beads, which effectively extends the shelf life of perishable products such as mushrooms by reducing microbial spoilage and weight loss, highlights their practical potential in sustainable food packaging. The synergistic interaction between the ZnO nanoparticles and the biopolymer matrix not only stabilized the enzyme but also enabled concurrent bioactivities within a single biodegradable system, offering an eco‐friendly alternative to synthetic preservatives. Furthermore, the beads demonstrated satisfactory reusability, supporting their economic viability for industrial‐scale applications. This work contributes a novel enzyme‐functionalized packaging approach that integrates catalytic efficiency, preservation function, and environmental compatibility. Future studies should investigate the system's performance under various storage conditions, assess its interaction with other bioactive agents, and evaluate its applicability to a broader range of food products and pathogens to validate its utility in the field of active and smart packaging technologies.

## Author Contributions

**Songul Bayrak:** investigation; methodology, conceptualization; data curation, visualization; formal analysis, writing – original draft; supervision, writing – review and editing.

## Conflicts of Interest

The author declares no conflicts of interest.

## Data Availability

Data supporting the findings of this study are available from the corresponding author upon request.

## References

[fsn370694-bib-0001] Abd Rahim, S. N. , A. Sulaiman , F. Hamzah , et al. 2013. “Enzymes Encapsulation Within Calcium Alginate‐Clay Beads: Characterization and Application for Cassava Slurry Saccharification.” Procedia Engineering 68: 411–417.

[fsn370694-bib-0002] Abdulaal, W. H. , Y. Q. Almulaiky , and R. M. El‐Shishtawy . 2020. “Encapsulation of HRP Enzyme Onto a Magnetic Fe3O4 np–PMMA Film via Casting With Sustainable Biocatalytic Activity.” Catalysts 10, no. 2: 181.

[fsn370694-bib-0003] Abo‐Dief, H. M. , O. K. Hussein , A. Ihsan , et al. 2022. “Ternary Metal Oxide WO3. NiO. ZnO Nanoparticles and Their Composite With CNTs for Organic Dye Photocatalytic Degradation.” Ceramics International 48, no. 15: 22228–22236.

[fsn370694-bib-0004] Alatawi, A. D. , L. W. Niessen , and J. A. Khan . 2020. “Efficiency Evaluation of Public Hospitals in Saudi Arabia: An Application of Data Envelopment Analysis.” BMJ Open 10, no. 1: e031924. 10.1136/bmjopen-2019-031924 PMC7045210 31932390

[fsn370694-bib-0005] Alizadeh Behbahani, B. , and A. A. Imani Fooladi . 2018. “Development of a Novel Edible Coating Made by Balangu Seed Mucilage and Feverfew Essential Oil and Investigation of Its Effect on the Shelf Life of Beef Slices During Refrigerated Storage Through Intelligent Modeling.” Journal of Food Safety 38, no. 3: e12443.

[fsn370694-bib-0006] Almaz, Z. 2023. “Investigation of Biological Activities of Various 1, 2, 3‐Triazole Compounds: Their Effects on Cholinesterase Enzymes, Determination of Antioxidant Capacity and Antimicrobial Activity.” Journal of Biochemical and Molecular Toxicology 37, no. 3: e23277. 36514839 10.1002/jbt.23277

[fsn370694-bib-0007] Almaz, Z. , A. Oztekin , N. Abul , et al. 2021. “A New Approach for Affinity‐Based Purification of Horseradish Peroxidase.” Biotechnology and Applied Biochemistry 68, no. 1: 102–113. 32060967 10.1002/bab.1899

[fsn370694-bib-0008] Alotaibi, A. N. , A. Al‐Dakhil , H. A. Alwabsi , I. O. Althobaiti , R. M. El‐Shishtawy , and Y. Q. Almulaiky . 2025. “Sustainable Synthesis of Alginate–Cobalt Ferrite Nanocomposites for Horseradish Peroxidase Immobilization: Enhanced Stability, Reusability, and Catalytic Efficiency.” Bioprocess and Biosystems Engineering 48: 1207–1219. 40268762 10.1007/s00449-025-03171-z

[fsn370694-bib-0009] Barbosa, G. S. d. S. , M. E. P. Oliveira , A. B. S. Dos Santos , O. C. Sánchez , C. M. F. Soares , and A. T. Fricks . 2020. “Immobilization of Low‐Cost Alternative Vegetable Peroxidase (*Raphanus sativus* L. Peroxidase): Choice of Support/Technique and Characterization.” Molecules 25, no. 16: 3668. 32806564 10.3390/molecules25163668 PMC7466051

[fsn370694-bib-0010] Baruwati, B. , D. K. Kumar , and S. V. Manorama . 2006. “Hydrothermal Synthesis of Highly Crystalline ZnO Nanoparticles: A Competitive Sensor for LPG and EtOH.” Sensors and Actuators B: Chemical 119, no. 2: 676–682.

[fsn370694-bib-0011] Batool, I. , M. Imran , A. Anwar , et al. 2024. “Enzyme‐Triggered Approach to Reduce Water Bodies' Contamination Using Peroxidase‐Immobilized ZnO/SnO2/Alginate Nanocomposite.” International Journal of Biological Macromolecules 254: 127900. 37931863 10.1016/j.ijbiomac.2023.127900

[fsn370694-bib-0012] Bayrak, S. , S. Gerni , C. Öztürk , et al. 2024. “Lactoperoxidase Inhibition of Celecoxib Derivatives Containing the Pyrazole Linked‐Sulfonamide Moiety: Antioxidant Capacity, Antimicrobial Activity, and Molecular Docking Studies.” Journal of Biochemical and Molecular Toxicology 38, no. 11: e70055. 39527602 10.1002/jbt.70055

[fsn370694-bib-0013] Bayrak, S. , C. Öztürk , Y. Demir , Z. Alım , and Ö. İ. Küfrevioglu . 2020. “Purification of Polyphenol Oxidase From Potato and Investigation of the Inhibitory Effects of Phenolic Acids on Enzyme Activity.” Protein and Peptide Letters 27, no. 3: 187–192. 31577197 10.2174/0929866526666191002142301

[fsn370694-bib-0014] Behbahani, B. A. , M. Noshad , and A. Vasiee . 2024. “The Effect of Plantago Major Seed Mucilage Fortified With Cell‐Free Supernatant (CFS) of Probiotic *Lactococcus lactis* NJ414 on Extending Shelf Life and Ensuring the Safety of Lamb Meat Slices Stored at 4°C.” LWT–Food Science and Technology 210: 116868.

[fsn370694-bib-0015] Bilal, M. , and H. M. Iqbal . 2019. “Lignin Peroxidase Immobilization on ca‐Alginate Beads and Its Dye Degradation Performance in a Packed Bed Reactor System.” Biocatalysis and Agricultural Biotechnology 20: 101205.

[fsn370694-bib-0016] Bilal, M. , Y. Zhao , T. Rasheed , and H. M. Iqbal . 2018. “Magnetic Nanoparticles as Versatile Carriers for Enzymes Immobilization: A Review.” International Journal of Biological Macromolecules 120: 2530–2544. 30201561 10.1016/j.ijbiomac.2018.09.025

[fsn370694-bib-0017] Bingol, Z. , H. Kızıltaş , A. C. Gören , et al. 2021. “Antidiabetic, Anticholinergic and Antioxidant Activities of Aerial Parts of Shaggy Bindweed (*Convulvulus betonicifolia* Miller Subsp.)–profiling of Phenolic Compounds by LC‐HRMS.” Heliyon 7, no. 5: e06986. 34027185 10.1016/j.heliyon.2021.e06986 PMC8129935

[fsn370694-bib-0018] Bradford, M. M. 1976. “A Rapid and Sensitive Method for the Quantitation of Microgram Quantities of Protein Utilizing the Principle of Protein‐Dye Binding.” Analytical Biochemistry 72, no. 1–2: 248–254. 942051 10.1016/0003-2697(76)90527-3

[fsn370694-bib-0019] Chung Ill Min, C. I. , H. J. Han Jae Gu , K. W. Kong Won Sik , et al. 2018. “Regional Discrimination of Agaricus Bisporus Mushroom Using the Natural Stable Isotope Ratios.” Food Chemistry 264: 92–100. 29853410 10.1016/j.foodchem.2018.04.138

[fsn370694-bib-0020] Chuysinuan, P. , T. Thanyacharoen , S. Techasakul , and S. Ummartyotin . 2018. “Electrospun Characteristics of Gallic Acid‐Loaded Poly Vinyl Alcohol Fibers: Release Characteristics and Antioxidant Properties.” Journal of Science: Advanced Materials and Devices 3, no. 2: 175–180.

[fsn370694-bib-0021] Dalal, S. , and M. N. Gupta . 2007. “Treatment of Phenolic Wastewater by Horseradish Peroxidase Immobilized by Bioaffinity Layering.” Chemosphere 67, no. 4: 741–747. 17140630 10.1016/j.chemosphere.2006.10.043

[fsn370694-bib-0022] Deshmukh, R. K. , and K. K. Gaikwad . 2024. “Natural Antimicrobial and Antioxidant Compounds for Active Food Packaging Applications.” Biomass Conversion and Biorefinery 14, no. 4: 4419–4440.

[fsn370694-bib-0023] Erdoğan, S. , N. D. Çakar , R. Ulucak , Danish , and Y. Kassouri . 2021. “The Role of Natural Resources Abundance and Dependence in Achieving Environmental Sustainability: Evidence From Resource‐Based Economies.” Sustainable Development 29, no. 1: 143–154.

[fsn370694-bib-0024] Ge, X. , X. Huang , L. Zhou , and Y. Wang . 2022. “Essential Oil‐Loaded Antimicrobial and Antioxidant Zein/Poly (Lactic Acid) Film as Active Food Packaging.” Food Packaging and Shelf Life 34: 100977.

[fsn370694-bib-0025] Gerni, S. , and H. Özdemir . 2024. “Development of a New Affinity Chromatography Method for Purification of Horseradish Peroxidase Enzyme.” Biotechnology and Applied Biochemistry 71, no. 1: 202–212. 37904288 10.1002/bab.2532

[fsn370694-bib-0026] Gulcin, İ. 2020. “Antioxidants and Antioxidant Methods: An Updated Overview.” Archives of Toxicology 94, no. 3: 651–715. 32180036 10.1007/s00204-020-02689-3

[fsn370694-bib-0027] Gulcin, İ. 2025. “Antioxidants: A Comprehensive Review.” Archives of Toxicology 99, no. 5: 1–1997. 40232392 10.1007/s00204-025-03997-2 PMC12085410

[fsn370694-bib-0028] Gulcin, İ. , and S. H. Alwasel . 2022. “Metal Ions, Metal Chelators and Metal Chelating Assay as Antioxidant Method.” PRO 10, no. 1: 132.

[fsn370694-bib-0029] Gulmez, C. , C. Altinkaynak , N. Özdemir , and O. Atakisi . 2018. “Proteinase K Hybrid Nanoflowers (P‐hNFs) as a Novel Nanobiocatalytic Detergent Additive.” International Journal of Biological Macromolecules 119: 803–810. 30077667 10.1016/j.ijbiomac.2018.07.195

[fsn370694-bib-0030] Hafeez, R. , J. Guo , T. Ahmed , et al. 2024. “Integrative Transcriptomic and Metabolomic Analyses Reveals the Toxicity and Mechanistic Insights of Bioformulated Chitosan Nanoparticles Against Magnaporthe Oryzae.” Chemosphere 356: 141904. 38582174 10.1016/j.chemosphere.2024.141904

[fsn370694-bib-0031] He, J. , G. Goksen , X. Cong , M. R. Khan , N. Ahmad , and W. Zhang . 2025. “Development and Characterization of Zein Co‐Encapsulated Wampee Essential Oil and Propolis Extract Films for Food Preservation.” Food Control 168: 110855.

[fsn370694-bib-0032] Hermanová, S. , M. Zarevúcká , D. Bouša , M. Pumera , and Z. Sofer . 2015. “Graphene Oxide Immobilized Enzymes Show High Thermal and Solvent Stability.” Nanoscale 7, no. 13: 5852–5858. 25757536 10.1039/c5nr00438a

[fsn370694-bib-0033] Imam, H. T. , P. C. Marr , and A. C. Marr . 2021. “Enzyme Entrapment, Biocatalyst Immobilization Without Covalent Attachment.” Green Chemistry 23, no. 14: 4980–5005.

[fsn370694-bib-0034] Kalsoom, U. , N. Khalid , A. Ibrahim , et al. 2023. “Biocatalytic Degradation of Reactive Blue 221 and Direct Blue 297 Dyes by Horseradish Peroxidase Immobilized on Iron Oxide Nanoparticles With Improved Kinetic and Thermodynamic Characteristics.” Chemosphere 312: 137095. 36334735 10.1016/j.chemosphere.2022.137095

[fsn370694-bib-0035] Köksal, Z. , Z. Alım , S. Bayrak , İ. Gülçin , and H. Özdemir . 2019. “Investigation of the Effects of Some Sulfonamides on Acetylcholinesterase and Carbonic Anhydrase Enzymes.” Journal of Biochemical and Molecular Toxicology 33, no. 5: e22300. 30811749 10.1002/jbt.22300

[fsn370694-bib-0036] Kumar, S. , A. Shukla , P. P. Baul , A. Mitra , and D. Halder . 2018. “Biodegradable Hybrid Nanocomposites of Chitosan/Gelatin and Silver Nanoparticles for Active Food Packaging Applications.” Food Packaging and Shelf Life 16: 178–184.

[fsn370694-bib-0037] Lahlali, R. , O. Mchachti , N. Radouane , et al. 2020. “The Potential of Novel Bacterial Isolates From Natural Soil for the Control of Brown Rot Disease (*Monilinia fructigena*) on Apple Fruits.” Agronomy 10, no. 11: 1814.

[fsn370694-bib-0038] Lavery, C. B. , M. C. MacInnis , M. J. MacDonald , et al. 2010. “Purification of Peroxidase From Horseradish (*Armoracia rusticana*) Roots.” Journal of Agricultural and Food Chemistry 58, no. 15: 8471–8476. 20681636 10.1021/jf100786h

[fsn370694-bib-0039] Liu, J.‐J. , J.‐G. Kim , H.‐B. Kim , S. Abeysinghe , Y.‐W. Lin , and K. Baek . 2023. “Covalent Immobilizing Horseradish Peroxidase on Electrochemically‐Functionalized Biochar for Phenol Removal.” Chemosphere 312: 137218. 36370757 10.1016/j.chemosphere.2022.137218

[fsn370694-bib-0040] Matto, M. , and Q. Husain . 2009. “Calcium Alginate–Starch Hybrid Support for Both Surface Immobilization and Entrapment of Bitter Gourd (*Momordica Charantia*) Peroxidase.” Journal of Molecular Catalysis B: Enzymatic 57: 164–170.

[fsn370694-bib-0041] Mohamed, J. A. , W. Huang , S. R. Nallapareddy , F. Teng , and B. E. Murray . 2004. “Influence of Origin of Isolates, Especially Endocarditis Isolates, and Various Genes on Biofilm Formation by *Enterococcus faecalis* .” Infection and Immunity 72, no. 6: 3658–3663. 15155680 10.1128/IAI.72.6.3658-3663.2004 PMC415661

[fsn370694-bib-0042] Mosallaie, F. , M. Pirnia , Z. Dehghan , et al. 2024. “Unveiling the Chemical Composition, Antioxidant and Antibacterial Properties, and Mechanistic Insights of *Convolvulus Arvensis* Extract Through Molecular Docking Simulations.” Applied Food Research 4, no. 2: 100580.

[fsn370694-bib-0043] Muhammad, W. , N. Ullah , M. Haroon , and B. H. Abbasi . 2019. “Optical, Morphological and Biological Analysis of Zinc Oxide Nanoparticles (ZnO NPs) Using *Papaver somniferum* L.” RSC Advances 9, no. 51: 29541–29548. 35531532 10.1039/c9ra04424h PMC9071912

[fsn370694-bib-0044] Muley, A. B. , P. Kedia , K. Pegu , S. B. Kausley , and B. Rai . 2022. “Analyzing the Physical and Biochemical Changes in Strawberries During Storage at Different Temperatures and the Development of Kinetic Models.” Journal of Food Measurement and Characterization 16: 222–226.

[fsn370694-bib-0045] Mustafa, F. , and S. Andreescu . 2020. “Nanotechnology‐Based Approaches for Food Sensing and Packaging Applications.” RSC Advances 10, no. 33: 19309–19336. 35515480 10.1039/d0ra01084g PMC9054203

[fsn370694-bib-0046] Naveenkumar, S. , S. Chandramohan , N. Alagumanikumaran , N. Venkateshan , K. Kaviyarasu , and A. Muthukumaran . 2024. “A Novel Occurrence of Polymorphic Self‐Assembled Zinc Oxide Nanoparticles Encapsulated by Sodium Alginate and Pectin.” Journal of Nanoparticle Research 26, no. 7: 149.

[fsn370694-bib-0047] Noshad, M. , B. A. Behbahani , and Z. Nikfarjam . 2022. “Chemical Composition, Antibacterial Activity and Antioxidant Activity of *Citrus Bergamia* Essential Oil: Molecular Docking Simulations.” Food Bioscience 50: 102123.

[fsn370694-bib-0048] Oleyaei, S. A. , Y. Zahedi , B. Ghanbarzadeh , and A. A. Moayedi . 2016. “Modification of Physicochemical and Thermal Properties of Starch Films by Incorporation of TiO2 Nanoparticles.” International Journal of Biological Macromolecules 89: 256–264. 27132884 10.1016/j.ijbiomac.2016.04.078

[fsn370694-bib-0049] Oztekin, A. , Z. Almaz , S. Gerni , et al. 2019. “Purification of Peroxidase Enzyme From Radish Species in Fast and High Yield With Affinity Chromatography Technique.” Journal of Chromatography B 1114: 86–92. 10.1016/j.jchromb.2019.03.035 30939412

[fsn370694-bib-0050] Öztürk, C. , S. Bayrak , Y. Demir , et al. 2022. “Some Indazoles as Alternative Inhibitors for Potato Polyphenol Oxidase.” Biotechnology and Applied Biochemistry 69, no. 5: 2249–2256. 34775655 10.1002/bab.2283

[fsn370694-bib-0051] Polat Kose, L. , and İ. Gulcin . 2021. “Evaluation of the Antioxidant and Antiradical Properties of Some Phyto and Mammalian Lignans.” Molecules 26, no. 23: 7099. 34885681 10.3390/molecules26237099 PMC8659077

[fsn370694-bib-0052] Saleem, M. S. , M. A. Anjum , S. Naz , et al. 2021. “Incorporation of Ascorbic Acid in Chitosan‐Based Edible Coating Improves Postharvest Quality and Storability of Strawberry Fruits.” International Journal of Biological Macromolecules 189: 160–169. 34411616 10.1016/j.ijbiomac.2021.08.051

[fsn370694-bib-0053] Salehi, M. M. , M. Mohammadi , A. Maleki , and E. N. Zare . 2024. “Performance of Magnetic Nanocomposite Based on Xanthan Gum‐Grafted‐Poly (Acrylamide) Crosslinked by Borax for the Effective Elimination of Amoxicillin From Aquatic Environments.” Chemosphere 361: 142548. 38852637 10.1016/j.chemosphere.2024.142548

[fsn370694-bib-0054] Salih, H. , W. Bai , Y. Liang , et al. 2024. “ROS Scavenging Enzyme‐Encoding Genes Play Important Roles in the Desert Moss *Syntrichia caninervis* Response to Extreme Cold and Desiccation Stresses.” International Journal of Biological Macromolecules 254: 127778. 37926320 10.1016/j.ijbiomac.2023.127778

[fsn370694-bib-0055] Sani, I. K. , S. Pirsa , and Ş. Tağı . 2019. “Preparation of Chitosan/Zinc Oxide/ *Melissa officinalis* Essential Oil Nano‐Composite Film and Evaluation of Physical, Mechanical and Antimicrobial Properties by Response Surface Method.” Polymer Testing 79: 106004.

[fsn370694-bib-0056] Shankar, S. , D. Khodaei , and M. Lacroix . 2021. “Effect of Chitosan/Essential Oils/Silver Nanoparticles Composite Films Packaging and Gamma Irradiation on Shelf Life of Strawberries.” Food Hydrocolloids 117: 106750.

[fsn370694-bib-0057] Shariat, S. Z. A. S. , F. Borzouee , M. R. Mofid , and J. Varshosaz . 2018. “Immobilization of Lactoperoxidase on Graphene Oxide Nanosheets With Improved Activity and Stability.” Biotechnology Letters 40: 1343–1353. 29915900 10.1007/s10529-018-2583-7

[fsn370694-bib-0058] Soliva‐Fortuny, R. C. , and O. Martín‐Belloso . 2003. “New Advances in Extending the Shelf‐Life of Fresh‐Cut Fruits: A Review.” Trends in Food Science & Technology 14, no. 9: 341–353.

[fsn370694-bib-0059] Talam, S. , S. R. Karumuri , and N. Gunnam . 2012. “Synthesis, Characterization, and Spectroscopic Properties of ZnO Nanoparticles.” International Scholarly Research Notices 2012, no. 1: 372505.

[fsn370694-bib-0060] Tanavar, H. , H. Barzegar , B. Alizadeh Behbahani , and M. A. Mehrnia . 2021. “Investigation of the Chemical Properties of *Mentha pulegium* Essential Oil and Its Application in *Ocimum basilicum* Seed Mucilage Edible Coating for Extending the Quality and Shelf Life of Veal Stored in Refrigerator (4 C).” Food Science & Nutrition 9, no. 10: 5600–5615. 34646530 10.1002/fsn3.2522 PMC8497838

[fsn370694-bib-0061] Thomas, D. , M. Latha , and K. K. Thomas . 2018. “Zinc‐Alginate Beads for the Controlled Release of Rifampicin.” Oriental Journal of Chemistry 34, no. 1: 428–433.

[fsn370694-bib-0062] Ton‐That, P. , T. A. Dinh , H. T. Gia‐Thien , N. Van Minh , T. Nguyen , and K. P. H. Huynh . 2025. “Novel Packaging Chitosan Film Decorated With Green‐Synthesized Nanosilver Derived From Dragon Fruit Stem.” Food Hydrocolloids 158: 110496.

[fsn370694-bib-0063] Urrea, D. A. M. , A. V. F. Gimenez , Y. E. Rodriguez , and E. M. Contreras . 2021. “Immobilization of Horseradish Peroxidase in ca‐Alginate Beads: Evaluation of the Enzyme Leakage on the Overall Removal of an Azo‐Dye and Mathematical Modeling.” Process Safety and Environmental Protection 156: 134–143.

[fsn370694-bib-0064] Vahidhabanu, S. , D. Karuppasamy , A. I. Adeogun , and B. R. Babu . 2017. “Impregnation of Zinc Oxide Modified Clay Over Alginate Beads: A Novel Material for the Effective Removal of Congo Red From Wastewater.” RSC Advances 7, no. 10: 5669–5678.

[fsn370694-bib-0065] Weber, A. C. , B. E. da Silva , S. G. Cordeiro , et al. 2023. “Immobilization of Commercial Horseradish Peroxidase in Calcium Alginate‐Starch Hybrid Support and Its Application in the Biodegradation of Phenol Red Dye.” International Journal of Biological Macromolecules 246: 125723. 37419265 10.1016/j.ijbiomac.2023.125723

[fsn370694-bib-0066] Weng, Y. , G. Yang , Y. Li , et al. 2023. “Alginate‐Based Materials for Enzyme Encapsulation.” Advances in Colloid and Interface Science 318: 102957. 37392664 10.1016/j.cis.2023.102957

[fsn370694-bib-0067] Xie, F. , M. Liu , X. Feng , et al. 2025. “Tannic Acid One‐Step Induced Quaternized Chitin‐Based Edible and Easy‐Cleaning Coatings With Multifunctional Preservation for Perishable Products.” Food Hydrocolloids 159: 110636.

[fsn370694-bib-0068] Yazıcıoğlu, Y. S. , Ş. Elmas , Z. Kılıç , et al. 2025. “Synthesis of Novel Isoindolinones: Carbonic Anhydrase Inhibition Profiles, Antioxidant Potential, Antimicrobial Effect, Cytotoxicity and Anticancer Activity.” Journal of Biochemical and Molecular Toxicology 39, no. 4: e70261. 40227019 10.1002/jbt.70261 PMC11995831

[fsn370694-bib-0069] Yildiz Dalginli, K. , and O. Atakisi . 2023. “Immobilization With ca–Alg@ Gelatin Hydrogel Beads Enhances the Activity and Stability of Recombinant Thermoalkalophilic Lipase.” Chemical and Process Engineering: New Frontiers 44, no. 1: 1–16. 10.24425/cpe.2023.142291.

[fsn370694-bib-0070] Yildiz Dalginli, K. , O. Atakisi , M. Ozturkler , and C. Gulmez Samsa . 2024. “Horseradish Peroxidase Immobilized in Calcium Alginate‐Gelatin Hybrid Beads With High Stability Against Metallic Ions and Organic Solvents.” Chemical Engineering Communications 211, no. 8: 1222–1235.

[fsn370694-bib-0071] Zanganeh, H. , S. A. Mortazavi , F. Shahidi , and B. Alizadeh Behbahani . 2021. “Evaluation of the Chemical and Antibacterial Properties of Citrus Paradise Essential Oil and Its Application in Lallemantia Iberica Seed Mucilage Edible Coating to Improve the Physicochemical, Microbiological and Sensory Properties of Lamb During Refrigerated Storage.” Journal of Food Measurement and Characterization 15, no. 6: 5556–5571.

[fsn370694-bib-0072] Zyoud, A. , A. H. Zyoud , S. H. Zyoud , et al. 2023. “Photocatalytic Degradation of Aqueous Methylene Blue Using Ca‐Alginate Supported ZnO Nanoparticles: Point of Zero Charge Role in Adsorption and Photodegradation.” Environmental Science and Pollution Research 30, no. 26: 68435–68449. 37126167 10.1007/s11356-023-27318-1

